# Effect of Androgen Deprivation on Long-term Outcomes of Intermediate-Risk Prostate Cancer Stratified as Favorable or Unfavorable: A Secondary Analysis of the RTOG 9408 Randomized Clinical Trial

**DOI:** 10.1001/jamanetworkopen.2020.15083

**Published:** 2020-09-09

**Authors:** Zachary S. Zumsteg, Daniel E. Spratt, Timothy J. Daskivich, Mourad Tighiouart, Michael Luu, Joseph P. Rodgers, Howard M. Sandler

**Affiliations:** 1 Department of Radiation Oncology, Cedars-Sinai Medical Center, Los Angeles, California; 2 Department of Radiation Oncology, University of Michigan, Ann Arbor; 3 NRG Oncology Statistics and Data Management Center, Philadelphia, Pennsylvania

## Abstract

This secondary analysis of a randomized clinical trial examines the effect of androgen deprivation therapy (ADT) during radiotherapy in patients who were classified as having either favorable intermediate-risk or unfavorable intermediate-risk prostate cancer.

## Introduction

Use of androgen deprivation therapy (ADT) during radiotherapy is controversial in intermediate-risk prostate cancer. Retrospective data suggest that ADT benefits patients with unfavorable, but not favorable, intermediate-risk cancer but are limited by selection bias and limited follow-up.^1,2^

## Methods

This study was an approved secondary analysis of NRG Oncology’s RTOG 9408 (ClinicalTrials.gov identifier: NCT00002597),^3^ a randomized clinical trial of radiotherapy with or without 4 months of ADT (see the full trial protocol in Supplement 1). All patients signed informed consent to enroll in the trial.

Pathology reports from 1068 intermediate-risk patients were reviewed by 3 physicians (Z.S.Z., D.E.S., and H.M.S.) to obtain the percentage of positive biopsy cores. Patients were stratified as having favorable intermediate-risk (FIR) and unfavorable intermediate-risk (UIR) prostate cancer according to primary Gleason score, percentage of positive biopsy cores, and number of intermediate-risk factors.^1^ One hundred seventy-eight patients (16%) were excluded because they could not be classified as FIR or UIR because of missing biopsy core information. Distant metastasis (DM) and prostate cancer–specific mortality (PCSM) were analyzed with competing-risks methods, and all-cause mortality (ACM) was assessed with the Kaplan-Meier method and Cox regression. Fifteen-year estimates are provided, but comparisons involved the entire survival curves. Restricted mean survival time analysis was also performed because the ACM curves were found to violate the proportional hazards assumption.^4^

Statistical analysis was performed using R statistical software version 3.5.1 (R Project for Statistical Computing) from November 2017 to May 2019. *P* values were calculated with 2-sided tests, and *P* <.05 was considered significant. See the eAppendix and the eFigure in Supplement 2 for additional methods.

## Results

The median follow-up duration was 17.8 years. A total of 890 patients (mean [SD] age, 70.3 [6.1] years) could be categorized as having either FIR (377 patients) or UIR (513 patients) cancer. Compared with patients classified as having FIR, patients classified as having UIR had higher risk of DM (hazard ratio [HR], 2.36; 95% CI, 1.44 to 3.89; *P* = .001), PCSM (HR, 1.84; 95% CI, 1.29 to 2.62; *P* = .001), and ACM (HR, 1.19; 95% CI, 1.02 to 1.40; *P* = .03) (Table). In patients with FIR, ADT did not improve DM (HR, 1.55; 95% CI, 0.64 to 3.74; *P* = .33), PCSM (HR, 0.63; 95% CI, 0.35 to 1.15; *P* = .13), or ACM (HR, 1.02; 95% CI, 0.80 to 1.30; *P* = .90) (Figure). By contrast, in patients with UIR, ADT improved DM (HR, 0.48; 95% CI, 0.28 to 0.83; *P* = .008) and PCSM (HR, 0.40; 95% CI, 0.26 to 0.60; *P* < .001) but not ACM (HR, 0.84; 95% CI, 0.68 to 1.03; *P* = .09). The 15-year restricted mean survival time was longer with ADT vs without ADT for patients with UIR (10.5 vs 9.8 years; difference, 0.7 year; 95% CI, 0.001 to 1.6 years; *P* = .0497), but there was no significant difference for patients with FIR (11.0 vs 10.7 years; difference, 0.3 year; 95% CI, −0.6 to 1.2 years; *P* = .50).

**Table.** Fifteen-Year Incidence of DM, PCSM, and ACM in Patients With FIR vs UIR Prostate Cancer Receiving ADT or Not

	15-y Incidence, %	HR (95% CI)^a^	*P* value
UIR vs FIR			
DM	17 vs 6	2.36 (1.44-3.89)	.001
PCSM	20 vs 11	1.84 (1.29-2.62)	.001
ACM	69 vs 61	1.19 (1.02-1.40)	.03
ADT vs no ADT			
DM: FIR	8 vs 5	1.55 (0.64-3.74)	.33
PCSM: FIR	9 vs 14	0.63 (0.35-1.15)	.13
ACM: FIR	62 vs 60	1.02 (0.80-1.30)	.90
DM: UIR	10 vs 24	0.48 (0.28-0.83)	.008
PCSM: UIR	12 vs 28	0.40 (0.26-0.60)	<.001
ACM: UIR	66 vs 71	0.84 (0.68-1.03)	.09

Abbreviations: ACM, all-cause mortality; ADT, androgen deprivation therapy; DM, distant metastasis; FIR, favorable intermediate-risk; HR, hazard ratio; PCSM, prostate cancer–specific mortality; UIR, unfavorable intermediate-risk.

^a^ HRs and 95% CIs are calculated with Cox regression for ACM and the Fine and Gray method for DM and PCSM.

**Figure.**
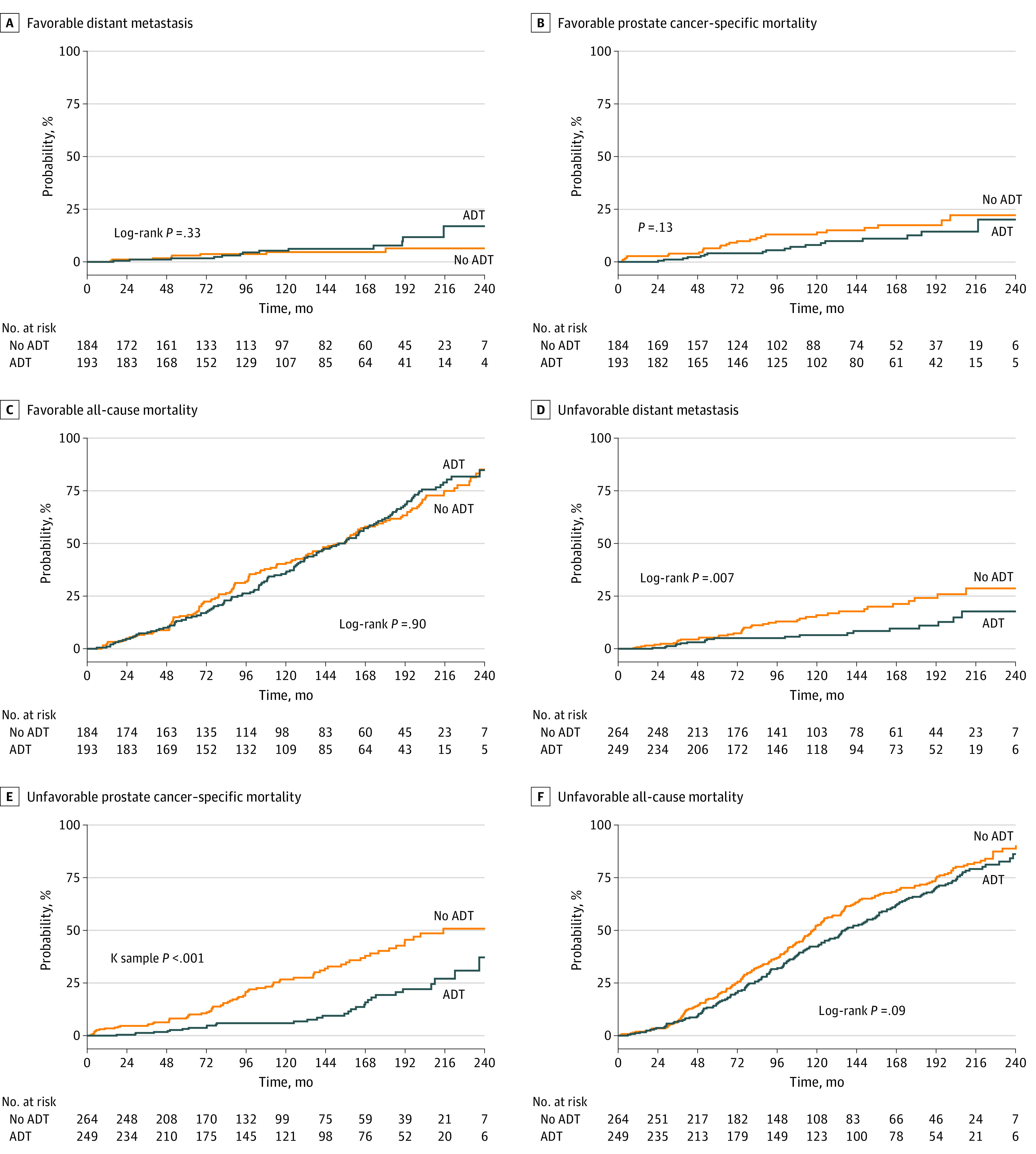
Outcomes for Patients With Favorable Intermediate-Risk or Unfavorable Intermediate-Risk Prostate Cancer Undergoing Radiation With or Without Androgen Deprivation Therapy (ADT) Panels A, B, and C show survival probabilities for patients with favorable intermediate risk (A, distant metastasis; B, prostate cancer-specific mortality; C, all-cause mortality), and panels D, E, and F show outcomes for patients with unfavorable intermediate-risk (D, distant metastasis; E, prostate cancer-specific mortality; F, all-cause mortality).

## Discussion

In this secondary analysis of the RTOG 9408 randomized clincial trial,^3^ FIR and UIR subclassifications were associated with higher risks of DM, PCSM, and ACM. Although previous studies have shown differences in prostate cancer–specific outcomes with FIR and UIR disease,^1,2,5^ to our knowledge, this study is the first to demonstrate an ACM difference.

Additionally, this study suggests that patients with UIR, but not FIR, undergoing radiotherapy have improved outcomes with short-term ADT. Although previous studies have also suggested this,^1,2^ they have been retrospective studies limited by selection bias in usage of ADT and short follow-up. Our study, using a large multi-institutional cohort from a cooperative group trial with randomized ADT use and nearly 18 years of follow-up, overcomes these limitations. To our knowledge, these are the highest-quality data supporting recent changes in the National Comprehensive Cancer Network guidelines recommending radiation without ADT for patients with FIR disease and combined ADT and radiotherapy for patients with UIR disease. Notably, given Gleason score inflation,^6^ improvements in radiation delivery, and advances in imaging over the last 25 years, it is likely that ADT would have even less benefit to contemporary patients with FIR than those enrolled in RTOG 9408.

Multiple limitations of this study warrant discussion, including that it is an unplanned secondary analysis and approximately 16% of patients with intermediate-risk cancer were excluded for having insufficient biopsy core information. Additionally, Gleason score migration over the last 2 decades and changes in radiation techniques make extrapolation to contemporary patients more challenging.

## Conclusions

In summary, to our knowledge, these results are the highest quality to date supporting a dichotomization of intermediate-risk prostate cancer into favorable and unfavorable subgroups, and support National Comprehensive Cancer Network recommendations to limit ADT use for patients with UIR disease. Future studies exploring genomic classifiers to further personalize therapy in intermediate-risk prostate cancer should be performed.

## Notes

Supplement 1. **Trial Protocol**

Supplement 2. **eAppendix.** Supplemental Materials and Methods **eReferences** **eFigure.** Consolidated Standards of Reporting Trials (CONSORT) of this Secondary Analysis of RTOG 9408

Supplement 3. **Data Sharing Protocol**
