# Supplementary material for: Effect of Androgen Deprivation on Long-term Outcomes of Intermediate-Risk Prostate Cancer Stratified as Favorable or Unfavorable: A Secondary Analysis of the RTOG 9408 Randomized Clinical Trial
Source: JAMA Netw Open. 2020 Sep 9;3(9):e2015083. doi: 10.1001/jamanetworkopen.2020.15083 (PMC7489808; doi:10.1001/jamanetworkopen.2020.15083)
Supplement: Supplement 1. — Trial Protocol [file jamanetwopen-e2015083-s001.pdf]

Supplementary Online Content

TRIAL PROTOCOL

RADIATION THERAPY ONCOLOGY

GROUP RTOG 94-08

A PHASE III TRIAL OF THE STUDY OF ENDOCRINE THERAPY USED AS A CYTOREDUCTIVE AND CYTOSTATIC AGENT PRIOR TO RADIATION THERAPY IN GOOD PROGNOSIS LOCALLY CONFINED ADENOCARCINOMA OF THE PROSTATE

Chairmen (11/17/05, 2/23/06, 7/14/08)

Radiation Oncology

David G. McGowan M.D.
(780) 432-8755
FAX # (780) 432-8380
davidmcg@cancerboard.ab.ca

Christopher U. Jones, M.D.
(916) 454-6600
FAX # (916) 454-6618
jonesC@radiological.com

Urology

Michael P. Chetner M.D.
(780) 484-4457
FAX # (780) 489-4549
mchetner@gpu.srv.ualberta.ca

Pathology

Mahul Amin, M.D.
(310) 423-6631
FAX # (310) 423-0170
aminm@cshs.org

Quality of Life

Deborah Watkins Bruner, R.N., Ph.D.
(215) 746-2356
FAX # (215) 573-7496
wbruner@nursing.upenn.edu

Activation Date: October 31, 1994

Update Date: February 23, 2006

Closure Date: April 30, 2001

Version Date: July 14, 2008

Includes Amendments 1-10
(Broadcast Date: September 30, 2008)

© 2020 Zumsteg ZS et al. *JAMA Network Open.*

This protocol was designed and developed by the Radiation Therapy Oncology Group *(RTOG)* of the American College of Radiology *(ACR)*. It is intended to be used only in conjunction with institution-specific IRB approval for study entry. No other use or reproduction is authorized by RTOG nor does RTOG assume any responsibility for unauthorized use of this protocol.

© 2020 Zumsteg ZS et al. *JAMA Network Open.*

INDEX

Schema
Eligibility Check
1.0 Introduction
2.0 Objectives
3.0 Patient Selection
4.0 Pretreatment Evaluations
5.0 Registration Procedures
6.0 Radiation Therapy
7.0 Drug Therapy
8.0 Surgery
9.0 Other Therapy
10.0 Pathology
11.0 Patient Assessments
12.0 Data Collection
13.0 Statistical Considerations
References

Appendix I - Sample Consent Form
Appendix II - Karnofsky Performance Status
Appendix III - Staging System
Appendix IV - Adverse Reaction Reporting Guidelines
Appendix V - Gleason Classification

© 2020 Zumsteg ZS et al. *JAMA Network Open.*

RADIATION THERAPY ONCOLOGY GROUP RTOG 94-08

A PHASE III TRIAL OF THE STUDY OF ENDOCRINE THERAPY USED AS A CYTOREDUCTIVE AND CYTOSTATIC AGENT PRIOR TO RADIATION THERAPY IN GOOD PROGNOSIS LOCALLY CONFINED ADENOCARCINOMA OF THE PROSTATE

SCHEMA

STRATIFY		RANDOMIZE	
	**PSA**<br>1. <4<br>2. ³ 4 *(to ≤20)*<br><br>**Grade** *(Differentiation)*<br>1. Well<br>2. Moderately<br>3. Poor<br><br>**Nodal Status**[c]<br>1. N0<br>2. NX		**Arm 1** Neoadjuvant TAS[a] 2 months before, and during RT[b]<br><br>**Arm 2** Radiation Therapy[b] Alone

a. Total Androgen Suppression (TAS): Patients will receive Flutamide *(two 125 mg capsules t.i.d., p.o.)* and Zoladex *(3.6 mg s.c. monthly x 4)* or Lupron, *(7.5 mg IM monthly x 4)* beginning two months before RT and continuing until RT is completed. Three-month depot preparation of Zoladex or Lupron may be used instead of three separate monthly injections.

b. Radiation : 46.8 Gy *(1.8 Gy/day four to five times a week [26 fx])* to regional lymphatics followed by 19.8 Gy *(1.8 Gy/day x 11 fx)* for a total of 66.6 Gy to the prostate. Prostate only may be treated in defined circumstances. See Section 6.4.2.

c. Nodal Status: Only nodes which are evaluated by surgical sampling will be classified as N0. Nodes evaluated negative by imaging methods only will be classified as NX.

ELIGIBILITY: *(See Section 3.0 for details)*

- Histologically confirmed locally confined adenocarcinoma of the prostate.
- Clinical stages T1b-2b.
- No involved nodes.
- No distant metastases.
- Karnofsky performance status ≥ 70.
- No prior antiandrogen therapy *(except as allowed by Section 3.1.5),* radiation or chemotherapy.
- Must sign a study-specific consent form.
- PSA is mandatory; *(PSA must be ≤ 20)*.
- Treatment on both arms must begin within 21 days after randomization
- No radical surgery or cryosurgery for prostate cancer

© 2020 Zumsteg ZS et al. *JAMA Network Open.*

Required Sample Size: 1980

4/1/95, 10/13/95, 4/5/96, 7/1/97, 9/8/98, 1/8/99, 11/13/00

© 2020 Zumsteg ZS et al. *JAMA Network Open.*

Institution # _______

RTOG 94-08 **ELIGIBILITY CHECK (11/13/00)**

Case #_____________ ***(page 1 of 2)***

______(Y) 1. Is there histologically confirmed locally confined adenocarcinoma of the prostate?

_____(T1b-2b) 2. What is the T stage? *(excluding T2 nos; must specify T2a or T2b)*

______(N0-NX) 3. What is the N stage?

______ (Y) If N0, was a surgical sampling done?

______(≥ 70) 4. What is the KPS?

______(≤ 20) 5. What is the PSA level?

______(N) 6. Has the patient had prior radiation or chemotherapy?

_____(N) 7. Has the patient had prior hormonal therapy *(excluding Proscar and testosterone)?*

______(N) 8. Has the patient had prior finasteride *(Proscar)* < 60 days and /or testosterone < 90 days before registration?

______(N) 9. Has the patient had prior radical surgery or cryosurgery for prostate carcinoma?

______(N) 10. Is there evidence of distant mets?

______(Y/NA) 11. Has the patient had a prior basal or squamous cell skin carcinoma and been NED for a minimum of two years?

______(Y/NA) 12. Has the patient had a prior malignancy *(other than Q11),* been disease free for ≥ 5 years, and been approved by the study chair?

______(Y) 13. Will treatment begin within the next 21 days?

______(N) 14. Are there any major medical or psychiatric illnesses that would prevent completion of treatment and interfere with followup?

______(Y) 15. Is the ALT < 2x your institution's upper normal limit?

(continued on next page)

© 2020 Zumsteg ZS et al. *JAMA Network Open.*

Institution # _______

RTOG 94-08 **<u>ELIGIBILITY CHECK</u> (11/13/00)**

Case #____________ ***(page 2 of 2)***

<u>The following questions will be asked at Study Registration:</u>

__________ 1. Name of institutional person registering this case?

(Y) 2. Has the Eligibility Checklist *(above)* been completed?

(Y) 3. Is the patient eligible for this study?

__________ 4. Date the study-specific Consent Form was signed? *(must be prior to study entry)*

__________ 5. Patient's Name

__________ 6. Verifying Physician

__________ 7. Patient's ID Number

__________ 8. Date of Birth

__________ 9. Race

__________ 10. Social Security Number

__________ 11. Gender

__________ 12. Patient's Country of Residence

__________ 13. Zip Code

__________ 14. Patient's Insurance Status

__________ 15. Will any component of the patient's care be given at a military or VA facility?

__________ 16. Treatment Start Date *(within 21 days after randomization)*

__________ 17. What is the combined Gleason Score?

__________ 18. PSA Value?

__________ 19. Grade *(Differentiation: well vs. moderately vs. poorly)*

__________ 2 N Stage?

© 2020 Zumsteg ZS et al. *JAMA Network Open.*

0.

____________ 2 Treatment Assignment

1.

Completed by____________________________________ Date ____________________

© 2020 Zumsteg ZS et al. *JAMA Network Open.*

1.0 INTRODUCTION

1.1 Endocrine hormonal therapy was introduced by Huggins in 1941[1] and is based on the dependence of prostatic epithelial cells and prostatic adenocarcinoma cells on androgenic hormones. Androgen deprivation by orchidectomy, estrogens or other antiandrogens can produce dramatic and often prolonged improvement in a large percentage of patients.

1.2 In early stage prostate cancer,[2,3] there is ample evidence that external beam radiation therapy is effective in the local control and cure of a large proportion of patients *(5 year disease free survival approximately 80% and at 10 years approximately 60%)*. Despite these results, approximately 30% of patients who are in complete clinical remission will have abnormal PSA's suggesting that these patients will eventually fail.

1.3 The use of a combination of cytoreductive hormonal therapy prior to and during radiation therapy has been the subject of several publications.[4,5,6] The recently completed RTOG study (RTOG 86-10) of locally advanced disease has demonstrated an improvement in disease-free survival *(at 2 years 76 % for hormones plus radiation therapy versus 62% for radiation therapy alone)*, and in addition a delay in relapse of approximately 9-12 months. There was also an improvement when PSA failure *(defined as a PSA greater than 4 g/L at 1-2.5 years)* was included. Since this study predated the introduction of PSA assays, data was not collected before treatment in all patients and, in addition, follow-up PSA data collection was incomplete.

1.4 Experimental data indicates that differentiated prostate cells undergo cell death in the absence of androgens[7] and that there is a significant killing of their stem cells. It has been postulated that the presence of androgens may suppress a cell death gene. The earliest apparent signs of morphologic change exhibited by the dying cells is described by a process called apoptosis. The cytoreductive effects of androgen deprivation may also result in the killing of a significant portion of prostate cancer stem cells and thus may contribute to the ability of radiation therapy to eradicate all the tumor stem cells at the primary site. The ability of androgen deprivation to improve local control with radiation therapy may actually be greater in small tumors than large tumors as the initial tumor stem cell population may be smaller and because smaller tumors are often more well differentiated.

1.5 A few publications[8,9,10] have appeared which suggest that PSA in excess of 4-5 times the upper limits of normal are associated with a low probability of cure with radiation therapy alone. For this reason a value of 20 has been set as the upper limit for this study. It may be considered by some ethically unwise to omit antiandrogen therapy in this group of patients. Conversely, patients with a PSA less than the upper limit of normal have a small risk of failure and thus the stratification.

1.6 Potential gains in survival must be weighed against potential losses in quality of life which in this particular protocol concern sexual function.

© 2020 Zumsteg ZS et al. *JAMA Network Open.*

Antiandrogenic treatment affects the central mechanisms mediating sexual activity. Whereas radiation therapy alone for prostate cancer may carry a 25-30% impotency rate,[20,21,22] hormonal therapy carries a risk of impotency of at least 80%.[22,23] The importance of sexual functioning cannot be taken lightly since Singer et al. were able to document a cohort of men with prostate cancer willing to trade potentially longer term survival options for treatment that offered a better chance of perserving potency.[24]

2.0 OBJECTIVES (7/14/08)

2.1 Primary Objectives

To evaluate whether a combination of Zoladex and flutamide used as cytoreductive agents prior to and during definitive radiation therapy improves overall survival over radiation therapy alone in locally confined carcinomas of the prostate

2.2 Secondary Objectives

2.2.1 To compare the rates of disease-specific survival, clinical relapse (local progression and/or distant failure), freedom from PSA failure, freedom from second clinical relapse, freedom from second PSA relapse, and disease-free survival

2.2.2 To compare the prostate re-biopsy at two years

2.2.3 To measure the effect on sexual function

© 2020 Zumsteg ZS et al. *JAMA Network Open.*

3.0 PATIENT SELECTION (9/8/98, 11/13/00)

3.1 Conditions for Patient Eligibility

3.1.1 Eligible patients will be those with histologically confirmed locally confined adenocarcinoma of the prostate. Included will be patients with primary tumors confined to the prostate, clinical stage T1b,1c, 2a or 2b.

3.1.2 Nodes evaluated negative by imaging methods will be classified as Nx. Nodes evaluated negative by surgical sampling will be classified as N0.

3.1.3 Karnofsky performance status ≥ 70.

3.1.4 PSA is mandatory for patient eligibility; *(PSA must be ≤ 20)*

3.1.5 No prior hormonal therapy, radiation or chemotherapy.

3.1.5.1 Prior finasteride for prostate hypertrophy is allowed if discontinued at least 60 days prior to randomization.

3.1.5.2 Prior testosterone administration is allowed if at least 90 days have elapsed since last administration *(testosterone effect will have receded by 90 days)*.

3.1.6 No evidence of distant metastasis or other synchronous primary. Patients with prior invasive malignancy who have been disease free for at least 5 years may be eligible. Before entry, these patients must be approved by the study chairman.

3.1.7 Treatment must begin within 21 days after randomization.

3.1.8 Patients must sign a study-specific informed consent form.

3.1.9 ALT must be within 2x upper normal limits.

3.2 Conditions for Patient Ineligibility (10/13/95)

3.2.1 Stage T1a or ≥ T2c disease.

3.2.2 Lymph node involvement *(N1 - N3)*.

3.2.3 Evidence of distant metastasis. *(M1)*

3.2.4 PSA > 20.

3.2.5 Radical surgery or cryosurgery for carcinoma of the prostate, previous irradiation, antiandrogen therapy or chemotherapy.

3.2.6 Previous or concurrent cancers other than basal cell or squamous cell skin carcinoma. Patients with squamous cell carcinomas must be NED for a minimum of two years prior to study entry.

3.2.7 Major medical or psychiatric illness which, in the investigator's opinion, would prevent completion of treatment and would interfere with follow-up.

3.2.8 Karnofsky performance status of < 70.

4.0 PRETREATMENT EVALUATION (7/1/97, 9/8/98, 11/13/00, 7/14/08)

4.1 History, physical examination *(to include tumor measurements)* and Karnofsky performance status.

4.2 Sexual history *(assessment of potency status)* is mandatory.

4.3 Histological evaluation. Tumors must be graded *(well-differentiated, moderately differentiated, or poorly differentiated)*. Gleason scores *(Appendix V)* should be provided when possible.

4.4 Mandatory laboratory studies: CBC, ALT, serum testosterone levels, alkaline phosphatase and a prostatic-specific antigen *(PSA)* study are mandatory for all patients.

© 2020 Zumsteg ZS et al. *JAMA Network Open.*

4.5 Bone scan *(mandatory)*.

4.6 Lymph node evaluation is mandatory and can be performed by at least one of the following: lymphangiogram, CT of the pelvis or exploratory laparotomy or laparoscopy with lymph node biopsy *(sampling)*.

5.0 REGISTRATION PROCEDURES

5.1 Patients can be registered only after pretreatment evaluation is completed and eligibility criteria are met. Patients are registered prior to any protocol therapy by calling RTOG headquarters at *(215)* 574-3191, Monday through Friday 8:30 am to 5:00 pm ET. The patient will be registered to a treatment arm and a case number will be assigned and confirmed by mail. The following information must be provided:

- Institution Name & Number
- Patient's Name & ID Number
- Verifying Physician's Name
- Eligibility Criteria Information
- Stratification Information

© 2020 Zumsteg ZS et al. *JAMA Network Open.*

- Demographic Data
- Treatment Start Date **(must be within 21 days of randomization)**

6.0 RADIATION THERAPY

6.1 Arm 1: In those patients randomized to Arm 1 *(drug Rx)*, radiation will start two months following initiation of drug administration.

6.1.1 *Total Androgen Suppression (TAS)*: Patients will receive Flutamide *(two 125 mg capsules t.i.d., p.o.)* and Zoladex, *(3.6 mg s.c. monthly x 4)*, or Lupron *(7.5 mg IM monthly x 4)* beginning 2 months before RT *(see Section 6.2.1)* and continuing until RT is completed. **(11/13/00)**

6.2 Arm 2: In Arm 2 *(radiation therapy only)* treatment will start without delay after randomization but no later than 21 days after randomization.

6.2.1 *Radiation*: 46.8 Gy *(1.8 Gy/day four to five times a week [26 fx])* to regional lymphatics followed by 19.8 Gy *(1.8 Gy/day x 11 fx)* for a total of 66.6 Gy to the prostate *(prostate only, in defined circumstances, see Section 6.4.2)*. **(4/5/96, 7/1/97)**

6.3 Physical Factors

Megavoltage equipment is required with effective photon energies ≥ 4 MeV *(≥6 MeV is preferable)*. Minimum source-to-axis distance is 100 cm. The minimum source-to-skin distance is 80 cm. Any treatment technique *(field arrangement)* capable of producing the dose distribution specified by the protocol will be acceptable with the exception of a perineal boost.

6.4 Target Volumes (4/5/96, 7/1/97)

6.4.1 In order to evaluate the adequacy of field margins, it is essential that the submitted simulator films used for defining the treatment volume contain contrast material in the bladder and rectum. If only CT planning is used, a copy of the planning CT must be submitted.

6.4.2 *Prostate*: The prostate only may be treated in those patients who meet the following criteria:

6.4.2.1 When a lymph node dissection is negative regardless of PSA value and Gleason score

OR

6.4.2.2 Patients with a PSA ≤ 10 AND Gleason score ≤ 5/10 *(or well differentiated)*.

6.4.2.3 All others will receive pelvic lymph node irradiation.

6.4.3 *Regional Lymphatics Target Volumes*

Patients will be treated electively to a target volume to include pelvic nodes to the level of the L5-S1 interspace. The inferior margin of the pelvic target volume will be placed at least 5 cm *(but no more than 6 cm)* from the superior margin of the symphysis. The lateral margins will be 2 cm lateral to the pelvic brim.

An AP-PA technique is not acceptable except with > 24 MeV photons. If a four-field technique is used, care should be taken to adequately cover external and internal iliac node chains and extensions of the primary tumor into the seminal vesicles and/or perirectal tissues. To achieve these goals, a major part of rectum may need to be included in the lateral fields.

6.4.4 *Prostate Boost Target Volume* will include the whole prostate with sufficient margins to include potential microscopic extensions. The prostatic boost target volume will measure at least 9.0 cm in longitudinal *(craniocaudal)* diameter and at least 8.0 cm in transverse and sagittal diameter. The seminal vesicles in their entirety are not considered to be target tissues. The size and the position of the prostatic boost target volume in these patients is optimally defined by the use of CT scan.

6.4.5 *Films*

Portal films of each treatment field and simulation films must be submitted to Headquarters for review.

6.5 Doses (7/1/97)

6.5.1 Daily tumor doses will be 1.8 Gy given once a day four to five times a week. The prescribed doses are defined on the central axis at the

© 2020 Zumsteg ZS et al. *JAMA Network Open.*

projected center of the target volumes. All patients will require isodose plans in the central plane of both the pelvic and prostate treatment volumes.

6.5.1.1 For two opposed coaxial equally weighted beams: on the central ray at mid-separation of beams.

6.5.1.2 For an arrangement of two or more intersection beams: at the intersection of the central ray of the beams.

6.5.1.3 For complete rotation or arc therapy: in the plane of rotation at the center of rotation.

6.5.1.4 For two opposing coaxial unequally weighted beams: on the central ray at the center of the target area.

© 2020 Zumsteg ZS et al. *JAMA Network Open.*

6.5.1.5 Other or complex treatment arrangements: at the center of the target area *(Note: there may be several target areas)*.

6.5.2 Regional lymphatics will receive a total of 46.8 Gy.

6.5.3 The prostatic target volume will receive a boost of 19.8 Gy bringing the total prescribed dose to that volume to 66.6 Gy in 37 fractions.

6.5.4 *Prostate radiation only*: The tumor dose will be 68.4 Gy at 1.8 Gy/fraction. This apparent discrepancy in dosage will arise from the premise that the whole prostate is encompassed by the 100% isodose in the pelvic field. If the prostate is not encompassed by the 100% isodose in the pelvic fields, adjustments in dosage will be required.

6.5.5 The minimal target dose to the regional lymphatics in the central plane will be 44 .4 Gy.

6.5.6 The minimal target dose to the prostatic target volume will be 65 Gy.

6.5.7 The permitted dose variation result in the target volume will be ± 5%.

6.5.8 The maximal target dose defined as the greatest dose in target volume which is delivered to an area greater than 2 cm^2 shall be 50 Gy for the regional lymphatics target volume and 70 Gy for the prostate boost target volume.

6.6 Critical Normal Structures

6.6.1 The bladder will receive the same dose as the regional lymphatics. Base of the bladder will be included in the prostate target volume and will receive the same dose as the prostate. Every attempt should be made to keep the bladder distended during administration of the boost in order to avoid irradiation of the superior portion of the organ.

6.6.2 Doses to the whole rectum shall not exceed 55 Gy. Portions of the anterior wall will, by necessity, receive the same dose as the prostate.

6.7 Radiation Toxicity (7/14/08)

6.7.1 All patients will be seen weekly by their radiation oncologist during radiation therapy. Any observations regarding radiation reactions will be recorded and should include attention toward the following potential side effects:

6.7.1.1 Skin reactions,

6.7.1.2 Small bowel or rectal irritation manifesting as abdominal cramping, diarrhea, rectal urgency, hematochezia,

6.7.1.3 Bladder complications including urinary frequency, dysuria, hematuria, urinary tract infections, and incontinence.

6.8.1.4 Impotence in previously potent patients.

7.0 DRUG THERAPY (ARM 1)

7.1* Zoladex *(NSC# 606864)

7.1.1 *Description*:

Zoladex is an LHRH analog with substitutions for the L-amino acid Glycine in positions 6 and 10. These substitutions produce an analog with 50-100 times the potency and longer duration of action than the naturally occurring peptide when assessed in acute animal tests.

7.1.2 *Supply*: **(7/1/97, 1/8/99)**

7.1.2.1 **Zoladex is commercially available.** For indigent patients who cannot afford the four months of neoadjuvant Zoladex, RTOG investigators may contact their local Zeneca sales representative to request compassionate use of Zoladex for an individual patient. Zeneca reserves the right to terminate this program for administrative reasons at any time with 30 days advance notice.

© 2020 Zumsteg ZS et al. *JAMA Network Open.*

7.1.2.2 Lupron 7.5 mg IM may be used instead of 3.6 mg Zoladex. See Section 7.3. If administered in place of Zoladex, this must be clearly specified on the RTOG flowsheets.

7.1.3 *<u>Preparation and Storage</u>*: **(1/8/99)**

The Zoladex depot is supplied preloaded with 3.6 mg Zoladex *(or 10.8 mg in the 3-month depot)* in a disposable syringe with a 16 gauge needle. The unit is sterile and comes in a sealed, light- and moisture-proof package.

The pack should be stored at approximately 25°C *(room temperature)*. Before being opened, each package must be inspected for damage in which case the syringe must not be used. Being sterile, the syringe should be removed from its package only by the physician/nurse immediately before needed.

7.1.4 *<u>Administration</u>*: **(1/8/99)**

Zoladex depot *(3.6 mg s.c.)* will be injected every four weeks beginning two months prior to radiation therapy. The 3-month preparation may be used instead of three separate monthly injections. All patients will receive four monthly injections of Zoladex with an overall treatment

© 2020 Zumsteg ZS et al. *JAMA Network Open.*

time of 112 days. If requested by the patient, a local anesthetic, i.e. 0.2 to 0.5 ml of 1% lidocaine hydrochloride may be given intradermally. Zoladex will be injected subcutaneously using an aseptic technique. Insert the needle to its full length, pull it back 1cm, then inject. The manufacturer recommends inserting the needle into the subcutaneous fat then changing the direction of the needle so it parallels the abdominal well before inserting the needle to its full length. This will create a little pocket for the Zoladex plug so that it does not extend when the needle is withdrawn. After checking to ensure that the depot has been discharged, the used syringe will be discarded in a safe manner. One can check to ensure that the depot has been discharged by ensuring the tip of the plunger is visible within the tip of the needle. The tear off portion of the depot package label will be removed and affixed to the appropriate data flowsheet as part of the patient's permanent record. In the event of radiotherapy treatment interruptions, the drug administration will be continued. Administration of drug will be suspended only if there is an apparent or suspected reaction to the drug.

7.1.5 *Toxicity*: (4/1/95)

During routine screening of Zoladex, no significant pharmacological activity was apparent in the cardiovascular, respiratory, central nervous, renal, metabolic, coagulation or gastric acid secretory systems. The acute toxicity of Zoladex has been found to be very low in relation to its pharmacological potency. Studies have shown that serum levels of testosterone can be reduced and maintained within the castrate range resulting in objective evidence of tumor regression. Other than the occasional transient worsening of cancer symptoms *(tumor flare)* due to an initial temporary rise in testosterone serum levels on initiating therapy, no significant toxicity apart from that attributed to castration *(hot flashes, decreased erections, impotence)* has been reported. Reports show that the incidence of localized or generalized rash with patients receiving Zoladex is 6%. There have been no reports of bronchospasm in the United States Clinical Trials program. In general, allergic reactions have been extremely uncommon with Zoladex therapy. There have been isolated reports of urethral obstruction, urticaria, or spinal cord compression. Shortness of breath, cardiac arrhythmia, hyperglycemia, severe back pain, acute kidney failure, pneumonia, confusion, weakness, pancreatitis and diabetes mellitus were reported in four men. No episodes of anaphylaxis as a result of Zoladex therapy have occurred in the past.

7.2 Flutamide *(NSC# 147834)*

7.2.1 *Description*:

Flutamide is a substituted anilide. It is a fine, light, yellow powder, insoluble in water but soluble in common organic solvents such as aromatic or halogenated hydrocarbons. Its concentration in plasma can be determined by gas chromatography. Flutamide is a non-steroid anti-androgen that is metabolized into a hydroxylated derivative which effectively competes with the hydrotestosterone for androgen receptor sites.

7.2.2 *Supply*:

Flutamide is commercially available.

7.2.3 *Storage*:

Flutamide is supplied as 125 mg capsules. Flutamide should be stored at temperatures ranging from 2°-30°C *(36°-86°F)* and be protected from excessive moisture.

7.2.4 *Administration*:

The drug is administered orally at a dose of two 125 mg capsules three times a day for a total daily dose of 750 mg beginning 2 months prior to radiotherapy and continuing throughout radiotherapy. Administration of flutamide will be terminated on the last day of radiotherapy or on day 112, whichever occurs first. During radiotherapy interruptions the drug will be continued. Administration of the drug will be suspended only if there is an apparent or suspected reaction to the drug.

7.2.5 *Toxicity*: (11/13/00)

The reported side effects of treatment include diarrhea and anemia. A high percentage of patients treated with flutamide alone developed gynecomastia within 2-8 months. There have been post-marketing reports of hospitalization, and, rarely, death due to liver failure in patients taking flutamide. Evidence of hepatic injury included elevated serum transaminase levels, jaundice, hepatic encephalopathy, and death related to acute hepatic failure. The hepatic injury was reversible after prompt discontinuation of therapy in some patients. Approximately half of the reported cases occurred within the initial 3 months of treatment with flutamide.

© 2020 Zumsteg ZS et al. *JAMA Network Open.*

7.2.6 *Dose Modification Schedule*: **(11/13/00)**

If gastrointestinal disturbances *(cramps, diarrhea)* occur prior to initiation of radiotherapy, flutamide will be withheld until the side effects subside and then reintroduced at a dose of 250 mg/day increasing the dose *(at 3 day intervals)* to 500 mg/day then 750 mg/day as tolerated.

If gastrointestinal disturbances occur after administration of radiotherapy it might be difficult to identify their cause. However if severity of diarrhea exceeds the level commonly observed during pelvic irradiation the toxicity will be ascribed to Flutamide and the drug will be permanently discontinued.

ALT will be measured pretreatment, then monthly during oral antiandrogen therapy. If ALT increases ≥ 2 x upper institutional limit of normal, flutamide must be discontinued. RTOG Headquarters must be notified.

7.3 Leuprolide (1/8/99)

7.3.1 *Description*

Leuprolide acetate is a synthetic nonapeptide analog of naturally occurring gonadotropin- releasing hormone *(GnRH or LH-RH)*. The analog possesses greater potency than the natural hormone. Leuprolide acetate, an LH-RH agonist, acts as a potent inhibitor of gonadotropin secretion when given continuously and in therapeutic doses. Animal and human studies indicate that following an initial stimulation, chronic administration of leuprolide acetate results in suppression of ovarian and testicular steroidogenesis. This effect is reversible upon discontinuation of drug therapy. Administration of leuprolide acetate has resulted in inhibition of the growth of certain hormone dependent tumors *(prostatic tumors in Noble and Dunning male rats and DMBA-induced mammary tumors in female rats)* as well as atrophy of the reproductive organs.

In humans, administration of leuprolide acetate results in an initial increase in circulating levels of luteinizing hormone *(LH)* and follicle stimulating hormone *(FSH)*, leading to a transient increase in levels of the gonadal steroids *(testosterone and dihydrotestosterone in males, and estrone and estradiol in premenopausal females)*. However, continuous administration of leuprolide acetate results in decreased levels of LH and FSH. In males, testosterone is reduced to castrate levels. In premenopausal females, estrogens are reduced to postmenopausal levels. These decreases occur within two to four weeks after initiation of treatment, and castrate levels of testosterone in prostatic cancer patients have been demonstrated for more than five years.

7.3.2 *Supply*

Leuprolide is commercially available as either 7.5 mg *(one month)*, 22.5 mg *(three month)*, or 30 mg *(four month)* depot injections. Each kit contains a vial of sterile lyophilized microspheres, which is leuprolide incorporated in a biodegradable polymer of polylactic acid.

7.3.3 *Storage*

The vial of leuprolide and the ampule of diluent may be stored at room temperature.

7.3.4 *Administration* **(11/13/00)**

Leuprolide will be administered *(7.5 mg IM monthly x 4 or 22.5 mg IM x 1 plus 7.5 mg IM x 1*). As with other drugs administered by injection, the injection site should be varied periodically. Incorporated in a depot formulation, the lyophilized microspheres are to be reconstituted and administered every three months as a single intramuscular injection.

7.3.5 *Toxicity*

In the majority of patients testosterone levels increased above baseline during the first week, declining thereafter to baseline levels or below by the end of the second week of treatment. The most common side effect of Leuprolide is vasomotor hot flashes; edema, gynecomastia, bone pain, thrombosis, and GI disturbances have occurred.

Potential exacerbations of signs and symptoms during the first few weeks of treatment is a concern in patients with vertebral metastases and/or urinary obstruction or hematuria which, if aggravated, may lead to neurological problems such as temporary weakness and/or paresthesia of the lower limbs or worsening of urinary symptoms.

7.4 Adverse Reaction Reporting (4/1/95, 1/8/99)

7.4.1 The following ADR's attributed to commercial agent(s) should be reported to the Investigational Drug Branch, Cancer Therapy Evaluation Program, within 10 working days:

7.4.1.1 Any ADR which is both serious *(life threatening, fatal)* and unexpected.

7.4.1.2 Any increased incidence of a known ADR which has been reported in the package insert or the literature.

7.4.1.3 Any death on study if clearly related to the commercial agent(s).

© 2020 Zumsteg ZS et al. *JAMA Network Open.*

7.4.1.4 The ADR Report should be documented on Form FDA 3500 and mailed to:

Investigational
Drug Branch Box
30012
Bethesda, MD 20824
(301) 230-2330 ***(24 hours)***
fax #301-230-0159

8.0 SURGERY

8.1 Pelvic Lymph Node Dissection

8.1.1 The pelvic lymph node dissection *(PLND)* should be done in order to accurately provide surgical/pathological staging for each patient randomized to RTOG 94-08. As suggested by previous RTOG protocols, surgical pathological staging has a dramatic impact on sub- stratification of patients and affects eventual outcome and survival statistics. The method by which the PLND is done, whether by a standard open procedure or by laparoscopic techniques, should not bias the study in any way. Both techniques of PLND, open and laparoscopic, are comparable in the literature in terms of the number of nodes removed and the incidence of positive nodes. There is an advantage to the laparoscopic PLND in that it lowers the length of stay in hospital and speeds patient convalescence and minimizes patient pain. It is an ideal form of surgical/pathological staging prior to definitive radiotherapy, allowing the radiotherapist to concentrate the fields on the prostate and periprostatic tissue and spare the pelvic lymph nodes *(assuming the dissection was negative for malignancy)*. Laparoscopic PLND may not be available at all institutions participating in RTOG 94-08, and therefore open surgical PLND is also acceptable.

8.2 Prostate Re-biopsy

8.2.1 The two-year biopsy will be performed for any patient who has achieved a clinical complete response *(NED)* or an equivocal disease response *(ED)* unless there is a medical contraindication to rebiopsy or definite evidence of local progression by clinical physical exam.

8.2.2 If the patient has distant metastatic disease or is post orchiectomy, the biopsy will not be performed.

9.0 OTHER THERAPY

Not applicable to this study.

10.0 PATHOLOGY (4/1/95, 7/1/97, 7/14/08)

10.1 Central pathology review of the diagnostic and of the 2-year biopsies are planned for this study. Central reviews of previous prostate studies have demonstrated a 34% discrepancy in histological grading.

© 2020 Zumsteg ZS et al. *JAMA Network Open.*

10.2 Hematoxylin and eosin*(H & E)* stained slides and a representative tissue block of all pathologic material, the pathology report and a pathology submission form will be submitted to:

Mailing Address: For Non-frozen Specimens Only
RTOG Biospecimen Resource
University of California San Francisco
Campus Box 1800
1657 Scott Street, Room 223
San Francisco, CA 94143-1800

Courier Address (FedEx, DHL, etc.): For Frozen Specimens
RTOG Biospecimen Resource
University of California San Francisco
1657 Scott Street, Room 223
San Francisco, CA 94115

Questions: 415-476-RTOG (7864)/FAX 415-476-5271; RTOG@ucsf.edu

10.2.1 H& E stained slides will be retained until completion of the analysis of the study. Slides will be returned if specifically requested at that time.

10.2.2 Blocks will be retained for the special studies outlined below. Slides prepared from the blocks will also be retained.

© 2020 Zumsteg ZS et al. *JAMA Network Open.*

10.2.3 If blocks will not be released, submission of 10 unstained sections mounted on sialinized *(or other "sticky slides")* may be substituted.

10.3 All pretreatment biopsies will be asssessed for the presence of tumor and graded according to Gleason *(see Appendix V)*.

10.4 DNA content and proliferation rate will be assessed in all cases by image analysis *(Feulgen staining)* and immunocytochemistry *(MIB-1 antibody).*

10.5 Pretreatment biopsies of patients randomized to receive hormone albation will be evaluated for expression of the bcl-2 proto-oncogene by immunohistochemistry. Overexpression of bcl-2 has been found to correlate with hormone resistance.[11,12] In this study, the potential value of bcl-2 as a predictor of hormone resistance will be studied.

10.6 Post-treatment biopsies will be assessed for the presence of persistent tumor.

10.6.1 All positive biopsies will be histologically graded according Gleason and the degree of therapy effect in the tumor cells will be graded according to Dhom and Degro.[13]

10.6.2 In cases where there is difficulty in diagnosis, immunohistochemical staining for high molecular weight cytokeratin will be performed to aid in the distinction of atypical benign glands from carcinoma.[14,15]

10.7 To encourage compliance, your Pathology Department can be reimbursed for obtaining blocks or cutting slides.

10.8 Patient consent form should give the Pathology Department authority and responsibility to comply with this request *(pathology blocks belong to the patient from whom tissue has been removed).*

11.0 PATIENT ASSESSMENTS

11.1 Study Parameters (10/1/96, 7/1/97, 9/8/98, 11/13/00)

Parameter	Pretreatment	At start of XRT	At end of XRT	Follow-up
History & Physical	X	X(b)	X	X
Karnofsky Status	X	X(b)	X	X
Sexual Status	X	X(b)	X	X
Chest X-ray				X(a)
Bone Scan	X			X(a)
CBC, alk. phos.	X	X(b)	X	X(e)
ALT	X	X(b,f)	X(b)	X(c)
Serum Testosterone	X		X	X(d)
Lymph Node Assessment	X			X(a)
PSA	X	X(b)	X	X(g)
Prostate Biopsy	X			at 24 months

a. As indicated
b. Only in patients assigned to receive drug treatment, Arm 1.
c. If abnormal following administration of drugs.
d. Serum testosterone will be repeated at the time of the first follow-up in all patients. If abnormal, repeat until it returns to the normal range or at 2 years, whichever comes first. For patients assigned to Arm 1, serum testosterone levels will be done when Zoladex is terminated and at 6 and 12 months post termination.
e. CBC is done at every followup for 2 years after end of treatment; alkaline phosphatase is performed yearly.
f. Monthly during hormone therapy. If liver functions rise to ≥ 2 x normal, flutamide will be discontinued and Dr. McGowan notified.

© 2020 Zumsteg ZS et al. *JAMA Network Open.*

g. All PSA determinations *(minimum of q 6 months)* within a followup period must be reported on the followup forms (F1); include a statement as to whether hormonal therapy was initiated for PSA or other relapse.

11.2 Follow-up Schedule (10/1/96)

11.2.1 Every 3 months for the remainder of the first year.
11.2.2 Every 4 months during the second year.
11.2.3 Every 6 months for years 3-5 then annually the remainder of the patient's life.

© 2020 Zumsteg ZS et al. *JAMA Network Open.*

11.2.4 A bone scan will be performed on any patient who presents with complaints of bone pain that cannot be attributed to any intercurrent disease. Discretionary plain films may be needed to evaluate lesions seen on bone scan to confirm the diagnosis of metastatic disease.

11.2.5 The patient will be asked whether he is able to achieve an erection and/or ejaculation and if he is able to have sexual relations. This assessment must be done prior to the start of any treatment, prior to the start of radiation therapy *(Arm 1)*, at the end of treatment and at each follow-up visit through year 2.

11.2.6 The Sexual Adjustment Questionnaire *(SAQ)* will be given to the patient to complete every three months for the remainder of the first year, then every four months in the second year. No SAQ will be required beyond the second year. An additional questionnaire will be completed prior to starting XRT but <u>after</u> the induction phase of hormones is completed.

11.2.7 Because an endpoint in this study is tumor clearance and local control, a biopsy of the prostate will be obtained at 24 months following completion of radiation therapy.

11.3 Measurement of Effect/Response

Prostate/prostate tumor dimensions in cm should be calculated from physical exam and must be recorded on the diagrams found on the data collection forms for initial and follow-up evaluation of the patient. After study entry, disease activity evaluations will be made and recorded using the following criteria:

11.3.1 <u>*No Evidence of Disease (NED)*</u>*:* no clinical evidence of disease on digital rectal examination.

11.3.2 <u>*Equivocal Disease (ED)*</u>*:* this rating will be assigned under the following two circumstances.

- If abnormalities are present on the prostate digital rectal examination but are thought to be abnormal due to treatment and felt not to represent tumor.
- If clinical evidence of residual tumor is present but this has regressed from a previous examination *(initial registration)*

11.3.3 <u>*Progressive Disease*</u> *(PD)*: this rating will be assigned when there is clinical evidence in the prostate gland of disease progression or recurrence measured by a 25% or greater increase in the product of the two largest perpendicular diameters of the prostate. Only those patients with progressive disease on digital rectal examination will be scored as digital rectal examination failure. The time of failure will be backdated to the first occurence of equivocal disease after a prior normal examination or to the end of radiation therapy treatment if a normal digital rectal examination was never achieved.

11.3.4 *Time to* <u>*Disease-Free Survival*</u>: Time to the disease-free survival will be measured from the date of randomization to the date of documentation of progression (local progression, distant failure, and PSA failure) or until the date of death *(from other causes)*. **(7/14/08)**

11.3.5 <u>*Time to Complete Response (CR)*</u>: Time in months from randomization to documentation of no evidence of disease *(NED)*.

11.3.6 <u>*Time to PSA Failure*</u>: Time in months from randomization to PSA failure as defined below:

11.3.6.1 A rise in PSA on at least two consecutive occasions above the nadir. In patients who have been declared a PSA relapse, every effort should be made to withhold further Rx until clinical relapse is evident. When this impossible, the site of failure should be ascertained before instituting further Rx. This may necessitate a bone scan, CT and prostate biopsy.

© 2020 Zumsteg ZS et al. *JAMA Network Open.*

11.3.6.2 The rises in PSA must exceed 1ng/ml above the nadir.

11.3.6.3 Failure of the PSA to reach 4 ng/ml or less at 18 months.

11.3.7 *Time to Second PSA Failure*:
The time from randomization to second PSA failure as defined below *(after institution of relapse therapy)*:

11.3.7.1 A rise in PSA on at least two consecutive cases above the nadir *(after initiation of relapase therapy)*.

11.3.7.2 The rises in PSA must exceed 1 ng/ml above the nadir.

11.3.7.3 Failure of the PSA to reach 4 ng/L or less at 18 months.

11.3.8 *Time to Local Progression*: The time to progression will be measured from the date of randomization to the date of documented local progression as determined by clinical exam.

11.3.9 *Time to Distant Failure*:
The time to distant failure will be measured from the date of randomization to the date of documented metastatic disease.

11.3.10 *Time to Overall Survival*: **(7/14/08)**
The time to overall survival will be measured from the date of randomization to the date of death due to any cause. All patients will be followed for survival. Every effort should be made

© 2020 Zumsteg ZS et al. *JAMA Network Open.*

to document the cause of death. Post-mortem examination will be carried out when feasible and a copy of the final autopsy report sent to RTOG.

11.3.11 *Time to Disease Specific Survival:* **(7/1/97) (7/14/08)**

The time to disease specific survival will be measured from the date of randomization to the date of the following events.

a. The following will be considered as endpoints in assessing disease specific survival *(DSS)*, i.e., events:
   - Death certified as due to prostatic cancer.
   - Death due to complications of treatment, irrespective of the status of malignancy.
   - Death from unknown causes with active malignancy *(clinical or biochemical progression).*
   - Death from unknown causes with previously documented relapse *(either clinical or biochemical).*

b. The following will be considered as events that may be related to death from prostate cancer. They will be added into the previous definition for a separate analysis.
   - Death from unknown causes without reported relapse.
   - Death from other causes with previous documented relapse *(either clinical or biochemical)* but inactive at the time of death.

11.3.12 *Sexual Functioning*: **(Discontinued 7/1/97)** Sexual functioning will be measured with the Sexual Adjustment Questionnaire *(SAQ)* developed by Waterhouse and Metcalfe. The questionnaire rates most responses on a five point Likert-type patient self-rating scale and includes a pre-treatment questionnaire and a follow-up questionnaire. Five of the most relevant of the eight original subscales of techinques, relationship, and miscellaneous were deleted for lack of relevance to this study and to avoid overburdening the patient with extra questions. Test-retest reliability was determined separately for each subsection of the SAQ questionnaire using Pearson correlation coefficient. Maximum r values for each subsection fell between .5389 and .9374. The overall mean reliability was .6721. The mean of the maximum r values for each subsection was .7790. Construct validity was determined by comparing differences in mean score on the SAQ between 84 healthy subjects and 8 subjects with head and neck cancer. Persons with cancer were found to have significantly *($p \geq .05$)* lower scores on the subsections testing activity level and relationships. For the remaining subscales, over 95% of the mean scores for the healthy subjects are greater than the mean scores for cancer patients although the differences are not significant.

12.0 DATA COLLECTION (4/1/95, 7/1/97)

**Item**	**Due**
Demographic Form **(A5)**	Within 1 week of study entry
On Study Form **(I1)**	Within 2 weeks of randomization
Pathology Report **(P1)**	
Pathology Slides/Blocks **(P2)**	
Surgical Operative Report **(S2)***	
Surgical Pathology Report **(S5)***	
* If node dissection or sampling was performed	
+ Pretreatment Sexual Adjustment Questionnaire **(SA)**	
Preliminary Dosimetry Information:	Within 1 week of start of RT
RT Prescription *(Protocol Treatment Form)* **(T2)**	
Films *(simulation and portal)* **(T3)**	
Calculations **(T4)**	
Interim Report **(F9)** *(Arm 1)*	

© 2020 Zumsteg ZS et al. *JAMA Network Open.*

Hormone Flowsheet **(M1)** *(Arm 1)*

Radiotherapy Form **(T1)**	Within 1 week of RT end
<u>Final Dosimetry Information</u>:	
Daily Treatment Record **(T5)**	
Isodose Distribution **(T6)**	

Boost Films *(simulation and portal)* **(T8)**

© 2020 Zumsteg ZS et al. *JAMA Network Open.*

Hormone Flowsheet **(M1)**	Upon completion of hormone therapy, thereafter with each follow-up form for 6 mos. following completion of hormones.
Follow-up Form **(F1)**<br>, every 6 mos during years 3-5 then death.	Upon completion of radiotherapy, then every 3 mos during year 1, every 4 mos. during year 2 annually. Also at progression/relapse and at
+ Post Rx Sexual Adjustment Questionnaire **(SB)**	Pre RT *(Arm 1)*; upon completion of treatment, then q 3 months during year 1, q 4 months in year 2.
Pathology Report/Rebiopsy **(P1)**<br>Pathology Blocks/Rebiopsy **(P2)**	At 2 years post RT
Autopsy Report **(D3)**	As applicable.

+ Completed by the patient. **(QOL assessments discontinued 7/1/97).**

13.0 Statistical Considerations

13.1 Study Design

The objectives of the trial are described in Section 2.0. They can be met by a randomized comparison of two groups testing neoadjuvant total androgen suppression *(TAS)* before/during RT versus RT alone.

Treatment allocation will be done by randomized permuted blocks within strata to balance for prognostic factors. Stratification will be done by PSA *(< 4, 4-20)* and differentiation *(well, moderate, poor)*, and nodal status *(N0, NX)*.

13.2 Study Endpoints (7/14/08)

13.2.1 Primary Endpoint

Overall Survival (See section 11.3.10)

13.2.2 Secondary Endpoints

13.2.2.1 Disease-specific survival (See section 11.3.11)

13.2.2.2 Local progression (See section 11.3.8)

13.2.2.3 Distant failure (See section 11.3.9)

13.2.2.4 Freedom from PSA failure (See section 11.3.6)

13.2.2.5 Freedom from second clinical relapse (local progression and/or distant failure)

13.2.2.6 Freedom from second PSA relapse (See section 11.3.7)

13.2.2.7 Disease-free survival (See section 11.3.4)

13.2.2.8 Prostate re-biopsy at two years

13.2.2.9 Effect on sexual function (See section 11.3.12)

13.3 Sample size and accrual (7/14/08)

As of July 1, 1997, the sample size is 1980 (See section 13.7).

TAS is known to lower PSA but time to initial PSA failure may not be a good indicator of ultimate clinical outcome. If neoadjuvant TAS is successful, there should ultimately be a positive impact on cause-specific survival. Because of the expected mortality due to other causes in this population, an improvement in cause-specific survival may be difficult to detect if overall survival were used as the primary endpoint. Clearly identifying deaths due to prostate cancer is difficult because metastasis is usually to the bone rather than to a vital organ.

The primary endpoint for this trial upon which sample size is based is defined to be closely associated to death due to prostate cancer. Review of causes of death by the study chair will be necessary.

The following will be considered treatment failures:

1. Death certified as due to carcinoma of the prostate.
2. Cancer associated death.
3. Death due to complications of treatment irrespective of the status of the cancer.
4. Progressive metastatic disease while on any antitumor therapy.
5. Rising PSA while on any antitumor therapy.

© 2020 Zumsteg ZS et al. *JAMA Network Open.*

Average failure rates were obtained from other datasets with the recognition that they may not be identical with what might be expected from this population.

Death rate: The death rate from early stage prostate patients from RTOG 77-06 had an average annual death rate of 0.045. Fourteen of the 46 reported deaths were categorized as due to prostate cancer. The prostate cancer death rate and other cause death rate were found by multiplying 0.045 by 14/46 and *(46-14)*/46.

Rate of first failure: This was taken to be 0.12 *(communication W. Shipley)*.

Rate of second failure while on hormonal therapy: This was taken to be 0.24 *(communication W. Shipley)*.

The goal of the trial is to be able to detect a lowering of the failure rates by 33% with 90% power using a two-sided test. Using methods described by Lakatos,[16,17] **a total of N=670 patients are required** to be accrued uniformly over three years and followed for three additional years before final analysis of treatment failure. This includes an estimate of 10% unevaluable patients. The expected numbers of events on the two arms at the time of final analysis are 71 and 39.

The Sexual Adjustment Questionnaire *(SAQ)* has five subscales; desire, activity level, arousal, orgasm, and satisfaction, that will be used to assess the effect of treatment, changes in serum testosterone, and other patient characteristics on patient's perception of quality of sexual function. The SAQ will be applied pretreatment, post radiotherapy, every three months for the first year of follow-up, and every four months for the second year of follow-up. Because it is unknown when a critical or defining change in sexual function occurs or remains stable all eight follow-up questionnaires are required and necessary for comparison of groups. Submission of the initial SAQ on RTOG 91-16 was 90% and 67% on RTOG 90-20. The submission of the follow-up questionnaires ranges from 40-90%.

This study should have sufficient numbers of patients completing the two-year follow-up SAQ to find a difference of at least one in the mean responses between groups for any of the five subscales. The estimated variance and completion rates will impact the required sample size. Because there is no reliable estimate of the SAQ response variance and completion rates will vary these parameters will be estimated after 200 patients have been randomized to this trial. These estimates will be utilized to determine whether additional patients will be accrued to the SAQ component. The results of this analysis will be presented to the Data Monitoring Committee *(DMC)*.

Functional status, the ability to have an erection, will be measured with a *'yes/no'* response at baseline and at each follow-up. It is assumed that at least 50% of all patients will enter the study with the ability to have an erection and 70% of these patients on the RT alone arm will retain this function. If 300 patients that enter the trial with the ability to have an erection are analyzable at 2 years, then this trial will have at least 80% statistical power to detect at least a 15% *(absolute)* reduction in the retention of function in the RT+TAS arm, testing at the 0.05 *(one-sided)* level.

13.4 Analysis Plan (11/13/00) (11/17/05) (7/14/08)

Analyses of time to treatment failure will use the log-rank test and the Cox proportional hazards model. All hypothesis testing will be two-sided. Exploration of the effects of pretreatment prognostic factors such as stage, Gleason score and PSA will be done using the Cox proportional hazards model. There will also be exploratory analyses of the different reasons for treatment failure.

Interim accrual and morbidity reports will be done at least semi-annually prior to the RTOG group meeting. The report will describe accrual by institution and project completion of accrual. It will give the distribution of pretreatment characteristics and a tabulation of toxicities.

Three interim treatment comparisons shall be performed when we observe 29%, 60% and 80% of the 261 required maximum number of prostate cancer related deaths. The first interim analysis is projected to take place at the second year after the closure *(every patient has at least one year of follow-up)*. The second and third interim analyses are projected to take place at the fifth year *(4 years of follow-up)* and seventh year *(6 years of follow-up)* after the closure, respectively. For each of these interim analyses, toxicity, treatment delivery and efficacy statistics will be reported to RTOG DMC. The boundary for early stopping *(or the nominal significance level for the test*) will be computed based on the observed number of deaths according to the O'Brian-Fleming alpha spending function approach.[18,19] If the difference is highly significant, i.e., p value less than the

© 2020 Zumsteg ZS et al. *JAMA Network Open.*

nominal level, the responsible statistician will recommend to DMC that the study be written up for publication.

Analysis of SAQ will be performed on all patients that complete baseline questionnaires. The analysis will utilize analysis of variance, and repeated measures analysis of variance to examine differences in treatments. SAQ will be examined for responsiveness in follow-up and correlation to changes in serum testosterone. Analysis of SAQ by subgroups will be undertaken for groups with sufficient sample size.

The study was designed for three interim analyses of the primary endpoint, disease-specific survival (death due to prostate cancer) when we observe 29% (76), 60% (157) and 80% (209) of the 260 required maximum number of prostate cancer related deaths. D'Amico et al[28] showed a statistically significant improvement in disease-specific survival with six months of total androgen blockade given two months before, during and after radiation therapy in a similar patient population. Based on this result, the disease site and study chairs requested an early look at disease-specific survival. This request was presented at the RTOG Data Monitoring Committee (DMC) in June 2005. The RTOG DMC approved an early analysis of the primary endpoint. Thus the first interim analysis is now planned to be done with 66 deaths. The boundary for early stopping (or the nominal significance level for the test) will be a modified Haybittle-Peto boundary. The stopping boundary of each of the three interim analyses is 3.291 (for a nominal significance level of 0.001) and the boundary for the final analysis is 1.96 which makes the overall significance level 0.051.

All eligible patients who are randomized to the study will be included in the comparison of treatment arms (intent-to-treat analysis).

13.4.1. Analysis of the Primary Endpoint

The number of events is based on the specific primary hypothesis that the overall survival rate of Arm 1 is higher than that of Arm 2. The results from D'Amico's paper[29] with a similar patient group showed an 8-year overall survival rate of 61% in patients treated with RT only. Based on this result, we assume the control arm (i.e., Arm 2) will have an 8-year overall survival rate of 60%, which translates to a yearly hazard rate of 0.0639. The study is designed to show an absolute improvement of 7% in the 8-year overall survival rate in Arm 1 (i.e., an 8-year overall survival rate of 67%), which translates to a yearly hazard rate of 0.0501). Assuming an exponential distribution for overall survival, 716 deaths are required to detect a 21.6% relative reduction in the yearly death rate with 90% statistical power using a one-sided log-rank test[30,31] at the 0.025 significance level.

A final analysis will be performed when 716 deaths occur. The time to overall survival will be measured from the date of randomization to the date of the death due to any cause (see section 11.3.10). We assume that the distribution of overall survival for each arm is an exponential distribution. We want to show that the hazard rate of Arm 1 (λ1) will be lower than that of Arm 2 (λ2) in the overall survival distribution. The following hypotheses are tested.

$$H_0: \lambda 2 \le \lambda 1 \quad \text{vs.} \quad H_A: \lambda 2 > \lambda 1$$

The survival distribution of overall survival will be estimated by the Kaplan-Meier method.[32] This hypothesis will be tested using a log-rank test statistic at a significance level $\alpha = 0.025$. In addition, the Cox regression model [33] will be used to compare the treatment differences. Both unadjusted and adjusted hazard ratios and their respective 95% confidence interval will be computed. At least the treatment arm, the stratification variables (PSA, grade, and nodal status), age, and race (as appropriate) will be adjusted for in this analysis.

13.4.2 Time to Failure of Secondary Survival Endpoints

The time to disease-free survival (DFS) will be measured from the date of randomization to the date of events (see section 11.3.4). The survival distribution of DFS will be estimated by the Kaplan-Meier method.[32] We want to show that the hazard rate of Arm 1 (λ_1) will be lower than that of Arm 2 (λ_2) in the DFS distribution. This hypothesis will be tested using a log-rank test statistic at a significance level $\alpha = 0.025$. The treatment effect on the other types of failure (disease-specific survival, local progression, distant failure, PSA failure, second clinical relapse,

© 2020 Zumsteg ZS et al. *JAMA Network Open.*

second PSA relapse) may impact the observable measures of outcomes and other competing risks may dilute the sensitivity. We will use the cause-specific hazard rate[34,35] approach to consider the competing events. Freidlin and Korn[36] showed that the cause-specific hazard rate approach is better than other approaches (for example, the cumulative incidence method[37] in most cases. The log-rank test on the times to the specific type of failure, which considers the presence of competing events, will be used to test whether the survival/failure rates of these secondary endpoints in Arm 1 are better than that of Arm 2 for each hypothesis at a significance level of 0.025. In this approach, patients who experience other failure first are censored. To analyze whether treatment effect is independently associated with secondary survival endpoints while adjusting for other factors, a Cox proportional hazards regression model [33] was used for DFS and Fine and Gray's regression model[38] were used for the other types of secondary survival endpoints. Both unadjusted and adjusted hazard ratios and the respective 95% confidence interval will be computed. At least the treatment arm, the stratification variables (PSA, grade, nodal status), age, and race (as appropriate) will be adjusted for in this analysis.

13.4.3 Prostate Re-Biopsy at Two Years

Because an endpoint in this study is local progression, a biopsy of the prostate will be obtained at two years (24 months) following completion of radiation therapy. The rate of prostate re- biopsy at two years is defined as the proportion of patients whose results are positive among all eligible patients. This rate will be estimated in each arm separately.

13.4.4 Effect on Sexual Function

Analysis of the Sexual Adjustment Questionnaire (SAQ) will be performed on all patients that complete baseline questionnaires. The SAQ has five subscales; desire, activity level, arousal, orgasm, and satisfaction, that will be used to assess the effect of treatment, changes in serum testosterone, and other patient characteristics on patients' perception of quality of sexual function. The SAQ will be administered pretreatment, post radiotherapy, every three months for the first year of follow-up, and every four months for the second year of follow-up. The analysis will utilize analysis of variance, and repeated measures analysis of variance to examine differences in treatments. The SAQ will be examined for responsiveness in follow-up and correlation to changes in serum testosterone. Analysis of SAQ responses by subgroups will be undertaken for groups with sufficient sample size. Functional status, the ability to have an erection, will be measured with a 'yes/no' response at baseline and at each follow-up. A two- sample proportion test with a different standard deviation will be used at the significance level of 0.05.

13.4.5 Interim Report to Monitor the Study Progress

Interim reports with descriptive statistics will be prepared twice per year until the initial paper reporting the treatment results has been submitted. In general, the interim reports will contain information about the patient accrual rate with a projected completion date for the accrual phase, the compliance rate of treatment delivery with the distributions of important prognostic baseline variables, and the frequencies and severity of the adverse event by treatment arm. The interim reports will not contain the results of the treatment comparisons with respect to the primary endpoint and secondary endpoints.

13.5 Inclusion of Women and Minorities (4/1/96)

In conformance with the National Institute of Health *(NIH)* Revitalization Act of 1993 with regard to inclusion of women and minority in clinical research, we have considered the possible interaction between race and treatments. Analysis of three prior RTOG studies failed to show such a significant difference[25] and so this study was not designed to test for treatment differences within race. A sensitivity analysis was done with the targeted sample size by racial groups in testing for treatment differences. If 88% of the patients recruited in this study are white/other, the statistical power to detect a reduction by 33% in the failure rate *(defined in Section 13.2)* is .86. If 12% of the patients recruited in this study are Afro-Americans, the statistical power to detect a reduction by 33% in the failure rate is .20.

The analysis for reporting the initial treatment results will include treatment comparisons within each race for the failure rate *(defined in Section 13.2)* and for overall survival.

13.6 Overview (7/1/97)

When the study was originally designed, a surrogate endpoint, time to second prostate-specific antigen failure, for cause specific survival was used as the primary endpoint. Zietman et. al. have reported on this surrogate endpoint using patients treated at Massachusetts General Hospital

© 2020 Zumsteg ZS et al. *JAMA Network Open.*

Cancer.[26] The statistician responsible for RTOG prostate studies was a consultant to that paper. It should be noted that 95% of the patients had T3 primaries and the remaining 5% had T4 primaries in the previous series, while only patients with T1b-T2b tumor are eligible for the current study.

Since RTOG 94-08 opened to patient entries, some information about overall survival and cause specific survival has become available from other RTOG prostate studies. Two significant findings that are relevant to this study are the cause of death and the importance of the Gleason score.

- A considerable percentage of deaths are attributable to causes other than prostate cancer. This percentage varies with T-stages and Gleason score groups. As seen in the table below, the percent of deaths attributable to prostate cancer decreases with the lower stages and/or lower Gleason groups.

Cause of Death

RTOG Protocol	Median FU Years*	Gleason Groups**	Total Deaths	Prostate No. (%)	Other No.(%)	Unknown No. (%)
7706 (T1/T2)	9.75	combined	258	76(29%)	165(64%)	17(7%)
	10.23	2-6	186	49(26%)	123(66%)	14(8%)
	7.69	7-10	62	25(40%)	34(55%)	3(5%)
8531+8610 (T3, RT only)	4.51	combined	159	75(47%)	73(46%)	11(7%)
	4.93	2-6	27	8(30%)	18(67%)	1(3%)
	4.44	7-10	127	67(53%)	52(41%)	8(6%)

***** for all eligible patients in the study
****** centrally reviewed

- The positive prostate study RTOG 85-31 showed that adjuvant hormonal arm is significantly better than the control arm in all disease related endpoints such as local control and disease free survival. However, survival benefit was only demonstrated in a group of patients with centrally reviewed Gleason scores 8-10.[27]

One implication of these findings, which will be illustrated further later, is that a significant difference in absolute survival may be practically unattainable unless the treatment effect in disease specific survival is large. With this new information, the RTOG statistical unit felt that the protocol should be amended to incorporate these findings. In general, if neoadjuvant TAS is successful, there should be positive impact on disease specific survival. Thus, we modified the study to use disease specific survival as the primary endpoint with a median of 10 years follow- up, while the overall survival is considered as a secondary endpoint for the study. We also acknowledge that Gleason score is a significant risk factor and must be included in the design.

Other studies have shown a high rate of recurrence free survival for patients whose pre-treatment PSA was less than 4. We decided not to utilize this information in the sample size calculation because there are no long term results available in men with PSA less than 4. Furthermore, almost all published studies showed a high correlation between pre-treatment PSA and Gleason score.

© 2020 Zumsteg ZS et al. *JAMA Network Open.*

13.7 Study Endpoints (7/1/97) (7/14/08)

The primary endpoint for this trial is overall survival, on which the sample size is calculated (See Section 11.3.10 for the definition). The absolute survival with failure of death from all

© 2020 Zumsteg ZS et al. *JAMA Network Open.*

causes is a secondary endpoint. We also consider the time to second PSA failure defined in Section 11.3.7 as a secondary endpoint for the trial. The collection of these data allows us to examine the hypothesis that the second PSA failure is a surrogate for death from prostate cancer. With an adequate long period of follow-up, we should be able to study the relationship among these times to endpoints.

13.8 Sample Size Determination (7/1/97)

13.8.1 *Assumptions*

1. The long-term follow-up results of a similar patient population treated by RT alone are available from RTOG 77-06. The figures *(a)* and *(b)* show a smoothed estimate of yearly hazard for patients with Gleason score 2-6 and Gleason score 7-10, respectively. These estimates will be used as the yearly hazards for the control arm in the sample size calculation. From the figures, we may notice that for patients with Gleason score 2-6 the hazard of death from all causes is 3-4 times as high as the hazard of death from prostate cancer; and for patients with Gleason 7-10 this ratio is about 2.

© 2020 Zumsteg ZS et al. *JAMA Network Open.*

Figure A

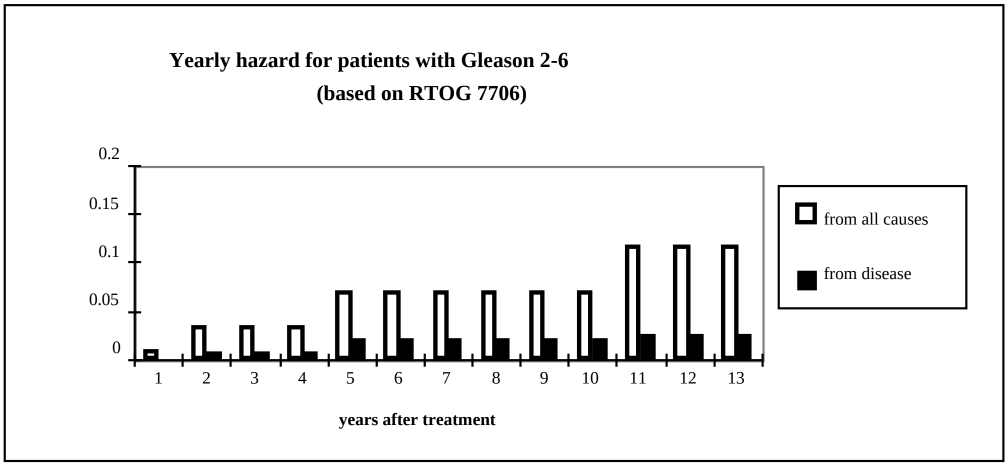

Figure B

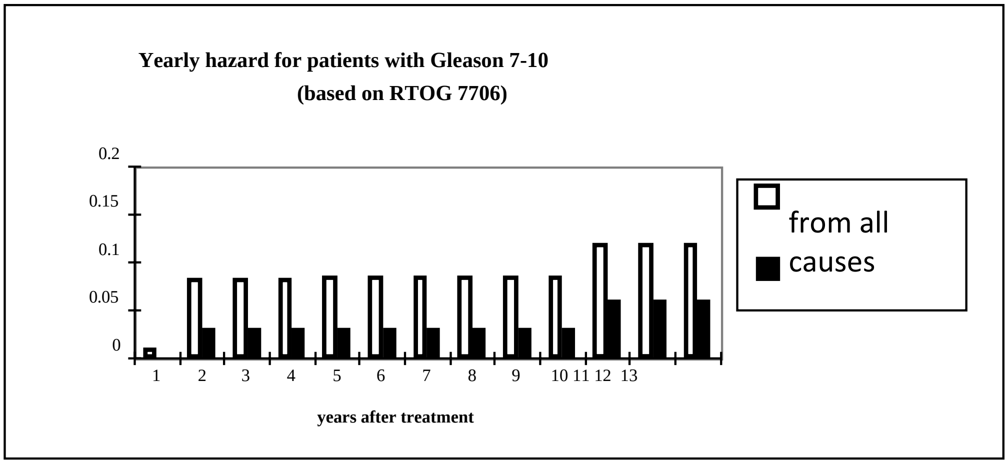

2. As of November 1996, 440 patients with pre-treatment data available have been entered into RTOG 94-08. Excluding ineligible patients and eligible patients without institutional Gleason scores, we estimate that 36% of the patients have Gleason scores of 7-10. *(As of May 1, 1997, this percentage is 38% with 622 patients having completed the initial evaluation)*.
3. To determine a reasonable estimate of the effect of neoadjuvant regimen, we have looked at a subpopulation of RTOG 8531 in which the patients mostly met the eligibility criteria of RTOG 9408 except for the criteria of the nodal involvement. We feel that the selected population may provide some insight about the population of RTOG 94-08 and its treatment effect. With 33 and 76 patients in Gleason 2-6 and Gleason 7-10 groups respectively, we calculated the effectiveness of RT plus immediate hormone assuming constant hazard. The following table lists hazard reduction in comparison to the control arm in overall survival and disease specific survival. Since only 5 years of follow up are available for RTOG 85-31 and few death

© 2020 Zumsteg ZS et al. *JAMA Network Open.*

© 2020 Zumsteg ZS et al. *JAMA Network Open.*

cases have been observed in patients with Gleason 2-6, only the estimated hazard reductions for the group of patients with Gleason 7-10 tumors can be used.

Estimated Hazard reduction in RTOG 8531

	Gleason 2-6	Gleason 7-10
Overall survival	8%	32%
Disease specific survival	NA	48%

We realize that because of the nodal involvement criteria of RTOG 8531, the selected sub-population includes less favorable patients than those in RTOG 94-08. Regardless of Gleason score groups, we would expect a hazard reduction in death from prostate cancer due to neoadjuvant hormone regimen to be less than 50%. Thus, we hypothesized a 33% hazard reduction in death from prostate cancer due to hormone treatment.

Based on the estimates of RTOG 77-06 given in assumption 1, we can translate the hazard reduction in disease specific survival into the hazard reduction in absolute survival. A 33% reduction in death from prostate cancer corresponds to an average of 11% reduction in death from all causes.

13.8.2 *Sample Size*

For the primary endpoint, disease specific survival, we will use a two-sided log-rank test to detect the treatment effect of 33% reduction in hazard of death from prostate cancer with a significance level of 0.05 and a power of 90%. Using Lakatos' method[16,17] *(assuming there is no drop-in and noncompliance, and loss of follow-up rate equals to the other cause mortality rate)*, a total of 1779 patients is *required to be accrued uniformly over five years and followed for additional eight years* before the final analysis. Accounting for possible 10% unevaluable cases, **the total required sample size is 1980 for both arms of the study.**

With the proposed sample size, we will be able to detect a 16.7% average reduction in hazard of death from all causes with 75% power. *(However, if the reduction in death from all causes is 11%, a formidably large sample size will be required)*.

If the current percentage *(36%)* of patients with Gleason 7-10 holds for the study, we will expect to enter 640 such evaluable patients. With this expected number of patients, we will be able to detect a 40% reduction in hazard of death from prostate cancer for patients with Gleason 7-10 with a statistical power of 80%. On the other hand, we will be able to detect a 20% reduction in hazard of absolute survival with statistical power of 53% for patients with Gleason 7-10.

© 2020 Zumsteg ZS et al. *JAMA Network Open.*

REFERENCES (7/14/08)

1. Huggins, C., Stevens, R.E., et al, Studies on Prostatic Cancer: The Effects of Castration on Advanced Carcinoma of the Prostate Gland. Arch. Surg. 43, 209, 1941.

2. Pilepich, M.V. Bagshaw, MA. et al. Definite Radiotherapy in Resectable (Stage A2 &B) Carcinoma of the Prostate - Results of a Nationwide Overview. Int. J. Rad. Onc Bio Phys 13:. 659-663, 1987.

3. McGowan, D.G. The Effect of Transurethral Resection on Prognosis in Carcinoma of the Prostate: Real or Imaginary? Int. J. R

4. Mukamelf, Servadio, C., Combined External Radiotherapy and Hormonal Therapy for Localized Carcinoma of the Prostate. Prostate 4, 283, 1983.

5. Pilepich, M.D., et al., Hormonal Cyto Reduction in Locally Advanced Carcinoma of the Prostate Treated with Definitive Radiotherapy Preliminary Results of RTOG 8307. Int. J. Rad. Onc. Bio. Phys. 16, 183, 1989.

6. Porter, A.T., Venner, P.M., The Role of Cyto Reduction Prior to Definitive Radiotherapy in Locally Advanced Prostate Cancer, 359, 231, 1990.

7. English, H.F., Kyprianov, N., Isaacs, J.T.. Relationship between DNA fragmentation and apoptosis in the programmed cell death in the rat following castration. Prostate 15: 233 - 250, 1989.

8. Russell, K.J., Dunatov, C et al. Prostate Specific Antigen in the Management of Patients with Localized Adenocarcinoma of the Prostate Treated with Primary Radiation Therapy. J. Urol 146: 1046 - 52, 1991

9. Zagars, G.K., Prostate-Specific Antigen as a Prognostic Factor for Prostate Cancer Treated by External Beam Radiotherapy. Int. J. Rad. Onc. Biol. Phys. 23: 47-53, 1992.

10. McGowan D.G. and Hanson, J., Prostatic Specific Antigen : An Independent Prognostic Factor in Adenocarcinoma of the Prostate Treated by External Beam Radiation (In Press)

11. Colombel M, Symmans F, Gil S, et al. Detection of the apoptosis-suppressing oncoprotein bcl-2 in hormone refractory human prostate cancers. Am J Pathol 143:390-400, 1993.

12. McDonnell TJ, Troncoso P, Brisbay SM, et al. Expression of the protooncogen bcl-2 in the prostate and its association with emergence of androgen-independent prostate cancer. Cancer Res 52:6940-6944, 1993.

13. Dhom G, Degro S. Therapy of prostatic cancer and histopathologic follow-up. Prostate 3:531-542, 1982.

14. Brawer MK, Nagle RB, Pits W, et al. Keratin immunoreactivity as an aid to the diagnosis of persistent adenocarcinoma in irradiated human prostates. Cancer 63:454-460, 1989.

© 2020 Zumsteg ZS et al. *JAMA Network Open.*

15. O'Malley FP, Grignon DJ, Shum DT. Usefulness of immunoperoxidase staining with high-molecular weight cytokeratin in the differential diagnosis of small acinar lesions of the prostate gland. Virchows Arch [A} 417:191-196, 1990.

16. Lakatos, E.: Sample size determination in clinical trials with time-dependent rates of losses and noncompliance. Controlled Clinical Trials 7:189-199, 1986.

17. Lakatos, E.: Sample sizes based on the log-rank statistic in complex clinical trials. Biometrics 44:229-241.

18. Kim K, and Tsiatis AA. Study duration for clinical trials with survival response and early stopping rule. Biometrics, 46: 81-92, 1990

19. O'Brien PC. and Fleming TR. A multiple testing procedure for clinical trials. Biometrics 35: 549-556, 1979.

© 2020 Zumsteg ZS et al. *JAMA Network Open.*

20. Banker, F.L.: The preservation of potency after external beam irradiation for prostate cancer. Int. J. Rad. Onc. Biol. Phys. 15: 219-220, 1988.

21. Ray, G.R., Cassady, J.R., Bagshaw, M.A. Definitive radiation therapy of carcinoma of the prostate: a report on 15 years of experience. Radiology 106: 407-418, 1973

22. Schover, L.R. Sexual rehabilitation after treatment for prostate cancer. Cancer 71 (3 Suppl): 1024-30, 1993.

23. Rousseau, L., Dupont, A., Labrie, F., Couture, M. Sexuality changes in prostate cancer patients receiving antihormonal therapy combining the antiandrogen flutamide with medical (LHRH Antagonist) or surgical castration. Archives Sexual Behavior. 17:87-98, 1988.

24. Singer, P., Tasch, E., Stocking, S., et al. Sex or survival: trade offs between quality and quantity of life. J. Clin. Oncol., 9: 328-334, 1991.

Formatted: English (U.S.)

*****25. Roach M, et al. The prognostic significance of race and survival from prostate cancer based on patients irradiated on Radiation Therapy Oncology Group protocols (1976-1985). Int J Rad Oncol Biol Phys 24: 441-449, 1992.

******26. Zietman, A. L., Dallow, K. C., et al, Time to Second Prostate-Specific Antigen Failure is a Surrogate Endpoint for Prostate Cancer death in a Prospective Trial of Therapy for Localized Disease. Urology 47: 236-239, 1996.

******27. Pilepich, M.V., Caplan, R., et. al., Phase III Trial of Androgen Suppression Using Goserelin in Unfavorable Prognosis Carcinoma of the Prostate Treated with Definitive Radiotherapy: Report of RTOG Protocol 85-31. J. Clinical Oncology, Vol. 15, No.3, 1997:pp1013-1021.

28. D'Amico, AV, Manola, J, Loffredo, M, Renshaw, AA, DellaCroce, A, Kantoff, PW. 6-Month Androgen Suppression Plus Radiation Therapy vs Radiation Therapy Alone for Patients With Clinically Localized Prostate Cancer: A Randomized Controlled Trial. JAMA 292(7): 821-827, 2004.

*******29. D'Amico AV. Chen M-H, Renshaw AA, et al. Androgen Suppression and Radiation vs. Radiation Alone for Prostate Cancer: A Randomized Trial, JAMA. 299(3), 289-295, 2008.

*******30. Mantel N. Evaluation of survival data and two new rank order statistics arising in its consideration. Canc Chemo Rep. 50:163-170, 1966.

*******31. Kim K, Tsiatis AA. Study duration for clinical trials with survival response and early stopping rule. Biometrics. 46:81-92, 1990.

*******32. Kaplan EL, Meier P. Nonparametric estimation from incomplete observations. J Am Stat Assoc. 53:447- 457, 1958.

*******33. Cox DR. Regression models and life tables. J Royal Statistics Soc. 34:187-220, 1972.

*******34. Kalbfleisch JD, Ross LP. The statistical analysis of failure time data. New York: John Wiley & Sons; 1980.

*******35. Gaynor JJ, Feuer EJ, Tan CC, et al. On the use of cause-specific failure and conditional failure probabilities: examples from clinical oncology data. JASA. 88:400-403, 1993.

*******36. Freidlin B, Korn EL. Testing treatment effects in the presence of competing risks. Stat Med. 24:1703- 1712, 2005.

******* 37. Gray RJ. A class of K-sample tests for comparing the cumulative incidence of a competing risk. Annual Statistics. 16:1141-1143, 1988.

© 2020 Zumsteg ZS et al. *JAMA Network Open.*

***38. Fine J, Gray RJ: A proportional hazards model for the sub-distribution of a competing risk. J Am Stat Assoc 94:496-509, 1999

* Added 4/5/96

**** Added 7/1/97**

***** Added 7/14/08**

© 2020 Zumsteg ZS et al. *JAMA Network Open.*

APPENDIX I

RTOG 94-08

A PHASE III TRIAL OF THE STUDY OF ENDOCRINE THERAPY USED AS A CYTOREDUCTIVE AND CYTOSTATIC AGENT PRIOR TO RADIATION THERAPY IN GOOD PROGNOSIS LOCALLY CONFINED ADENOCARCINOMA OF THE PROSTATE

SAMPLE PATIENT CONSENT FORM

RESEARCH STUDY

I have the right to know about the procedures that are used in my participation in clinical research so as to afford me an opportunity to make the decision whether or not to undergo the procedure after knowing the risks and hazards involved. This disclosure is not meant to frighten or alarm me; it is simply an effort to make me better informed so I may give or withhold my consent to participate in clinical research.

PURPOSE OF THE STUDY

It has been explained to me that I have prostate cancer. My doctor feels that my participation in this study may be helpful. This study involves the evaluation of drugs Zoladex and Flutamide used before and during a course of radiotherapy. The purpose of this study is to determine whether these drugs may improve the probability of tumor control when used in conjunction with radiotherapy.

DESCRIPTION OF PROCEDURES (4/1/95, 7/1/97, 1/8/99)

It is not clear at the present time which of the two regimens *(radiation with Zoladex and Flutamide or radiation alone)* is better. For this reason the therapy which is to be offered to me will be based upon a the method of selection called randomization. Randomization means that my physician will call a statistical office which will assign me one of the two regimens by computer. The chance of my receiving one of the two therapies is approximately equal. I will be assigned to one of these two treatments:

***Treatment 1*:** I will receive two monthly injections of Zoladex or Lupron under my upper abdominal skin, and six Flutamide capsules daily for two months. Then radiation will be given once a day, 4-5 days a week for almost eight weeks. The Zoladex *(or Lupron)* and Flutamide will be given on the same schedule during radiation as it was given before radiation began. Zoladex and Lupron can also be given as a three-month "time-release" injection instead of once a month.

***Treatment 2*:** Radiation will be given once a day, 4-5 days a week for almost eight weeks.

Also, at the time of my diagnosis by biopsy, all or some of my tumor was removed. As is usually done, this tissue went to the hospital's pathology department for routine testing and diagnosis. After that process was complete, remaining tumor samples were stored in the pathology department. I am being asked for permission to use the remainder of the tumor for additional tests. Since this tissue was removed at the time of surgery or biopsy, the permission to use my tissue will not involve any additional procedure or expense to me. The tumor tissue's cells will be examined to see if any special "markers", tests which predict how a patient with tumors like mine responds to treatment, can be identified.

At two years following radiotherapy, *(for both treatment regimens)*, I will be requested to have a biopsy of my prostate to microscopically evaluate the response to treatment.

© 2020 Zumsteg ZS et al. *JAMA Network Open.*

RISKS AND DISCOMFORTS (4/1/95, 7/1/97, 11/13/00)

Cancer treatments often have side effects. The treatment used in this program may cause all, some, or none of the side effects listed. In addition, there is always the risk of very uncommon or previously unknown side effects occurring. There is also the unknown risk that the delay before starting radiation therapy may allow the tumor to grow larger.

Zoladex and Lupron frequently lead to hot flashes, sexual dysfunction and decreased erections. A brief but temporary flare-up of tumor-related symptoms *(if any)* may also occur. The following have been reported as possible reactions *(1-5%)* to Zoladex: acute kidney failure, back pain, mental confusion, pressure on my spinal cord, spasms of the windpipe, chest pain, pneumonia, lung clots and cough or breathlessness, chills, fever, irregular heartbeat, elevated or low blood sugar, and weakness, anxiety, or depression. The relationship of these adverse reactions to hormone therapy is uncertain. The symptoms may reverse upon cessation of hormone treatment. Zoladex /Lupron may occasionally produce irritation at the site of injection. Very rarely, allergic reactions, generalized skin rash or vasculitis *(inflammation of the tissue beneath the skin)* has been reported.

Flutamide can cause impotence, loss of libido, breast tenderness, anemia, breast swelling, and hot flashes. The most frequently reported discomforts have been fatigue, back pain, and fluid retention. Approximately 2% of patients had constipation, diarrhea, or nausea or changes in liver function, though these are infrequent. Your liver function will be checked monthly while you are taking this agent. It is important to call your doctor immediately if you experience any of the following symptoms; intense itching, dark urine, loss of appetite, nausea and vomiting, yellow skin *(jaundice)* or eyes, abdominal tenderness or "flu-like" symptoms. There have been rare reports of death following severe liver damage from flutamide. Flutamide may cause photosensitivity. Avoid prolonged exposure to the sun and other ultraviolet light. Use sunscreens and wear protective clothing until tolerance is determined. Many of these changes improve or go away despite continuation of therapy.

Radiotherapy may cause reddening or tanning of the skin, hair loss in the treatment area, temporary fatigue, nausea, diarrhea, abdominal cramps, bladder irritation, and in some patients permanent impotence. There is also a small probability of injury to the bladder, urethra, bowel and other tissues in the pelvis or abdomen.

My physician will be checking closely to see if any of these effects are occurring. Appropriate tests will be done to monitor the effects of treatment. Appropriate medications will be prescribed to keep the side effects under control. I understand that the use of medications to help control side effects could result in added costs. This institution is not financially responsible for treatments for side effects caused by the study treatment.

CONTACT PERSONS

In the event that injury occurs as a result of this research, treatment will be available. I understand, however, I will not be provided with reimbursement for medical care other than what my insurance carrier may provide nor will I receive other compensation. For more information concerning the research and research-related risks or injuries, I can notify Dr. ________ the investigator in charge at ______________________________. In addition, I may contact ________________________at_______________________________for information regarding patients' rights in research studies.

BENEFITS

It is not possible to predict whether or not any personal benefit will result from the use of the treatment program. I understand that the information which is obtained from this study may be used scientifically and possibly be helpful to others. The possible benefits of this treatment program are greater shrinkage and control of my tumor and prolongation of my life but I understand this is not guaranteed.

I have been told that should my disease become worse, should side effects become very severe, should new scientific developments occur that indicate the treatment is not in my best interest, or should my physician feel that this treatment is no longer in my best interest, the treatment would be stopped. Further treatment would be discussed.

© 2020 Zumsteg ZS et al. *JAMA Network Open.*

ALTERNATIVES

Alternatives which could be considered in my case include radiation therapy alone, surgery, hormone therapy alone, treatment with Zoladex and Flutamide before and during radiation therapy but not on a study, chemotherapy or treatments to make me feel better, but not necessarily cure me or make my disease less. An additional alternative is no further therapy, which would probably, but not definitely, result in continued growth of my tumor. I understand that my doctor can provide detailed information about my disease and the benefits of the various treatments available. I have been told that I should feel free to discuss my disease and my prognosis with the doctor. The physician involved in my care will be available to answer any questions I have concerning this program. In addition, I understand that I am free to ask my physician any questions concerning this program that I wish in the future.

My physician will explain any procedures related solely to research. Some of these procedures may result in added costs but may be covered by insurance. My doctor will discuss these with me.

VOLUNTARY PARTICIPATION

Participation in this study is voluntary. No compensation for participation will be given. I understand that I am free to withdraw my consent to participate in this treatment program at any time without prejudice to my subsequent care. Refusal to participate will involve no penalty, or loss of benefits. I am free to seek care from a physician of my choice at any time. If I do not take part in or withdraw from the study, I will continue to receive care. In the event of a research-related injury, I understand my participation has been voluntary.

CONFIDENTIALITY

I understand that records of my progress while on the study will be kept in a confidential form at this institution and also in a computer file at the headquarters of the Radiation Therapy Oncology Group *(RTOG)*. The confidentiality of the central computer record is carefully guarded. During their required reviews, representatives of the Food and Drug Administration *(FDA)*, the National Cancer Institute *(NCI)*, qualified representatives of the drug manufacturers and other groups or organizations that have a role in the conduct of this study may have access to medical records which contain my identity. However, no information by which I can be identified will be released or published. Histopathologic material, including tissue and/or slides, will be sent to a central office for review and research investigation associated with this protocol. A representative tissue sample will be kept by the reviewing pathologist for additional tests. All samples and their associated information will be kept confidential.

I have read all of the above, asked questions, received answers concerning areas I did not understand, and willingly give my consent to participate in this program. Upon signing this form I will receive a copy.

Patient Signature *(or Legal Representative)* Date

© 2020 Zumsteg ZS et al. *JAMA Network Open.*

APPENDIX II

KARNOFSKY PERFORMANCE SCALE

100	Normal; no complaints; no evidence of disease
90	Able to carry on normal activity; minor signs or symptoms of disease
80	Normal activity with effort; some sign or symptoms of disease
70	Cares for self; unable to carry on normal activity or do active work
60	Requires occasional assistance, but is able to care for most personal needs
50	Requires considerable assistance and frequent medical care
40	Disabled; requires special care and assistance
30	Severely disabled; hospitalization is indicated, although death not imminent
20	Very sick; hospitalization necessary; active support treatment is necessary
10	Moribund; fatal processes progressing rapidly
0	Dead

© 2020 Zumsteg ZS et al. *JAMA Network Open.*

APPENDIX III

Prostate, AJCC 4th Edition, 1992

DEFINITION OF TNM

Primary Tumor **(T)**

TX Primary tumor cannot be assessed
T0 No evidence of primary tumor
T1 Clinically inapparent tumor not palpable or visible by imaging.
T1a Tumor incidental histologic finding in 5% or less of tissue resected
T1b Tumor incidental histologic finding in more than 5% of tissue resected
T1c Tumor identified by needle biopsy *(e.g., because of elevated PSA)*

T2 Tumor confined within prostate*
T2a Tumor involves half of a lobe or less
T2b Tumor involves more than half of a lobe but not both lobes.
T2c Tumor involves both lobes.

T3 Tumor extends through prostatic capsule** T3a
Unilateral extracapsular extension T3b
Bilateral extracapsular extension
T3c Tumor involves the seminal vesicle(s).

T4 Tumor is fixed or invades adjacent structures other than the seminal vesicles.
T4a Tumor involves any of: bladder neck, external sphincter, or rectum
T4b Tumor involves levator muscles and/or is fixed to the pelvic wall

*Note: Tumor found in one or both lobes by needle biopsy, but not palpable or visible by imaging, is classified as T1c
**Note: Invasion into the prostatic apex into *(but not beyond)* the prostatic capsule is not classified as T3, but as T2.

Regional Lymph Nodes **(N)**

NX Regional lymph nodes cannot be assessed
N0 No regional lymph node metastasis
N1 Metastasis in a single lymph node, 2 cm or less in greatest dimension
N2 Metastasis in a single lymph node, more than 2 cm but not more than 5 cm in greatest dimension, or multiple lymph nodes, metastases, none more than 5 cm in greatest dimension
N3 Metastasis in a lymph node more than 5 cm in greatest dimension

*Distant Metastasis** (M)

MX Presence of distant metastasis cannot be assessed
M0 No distant metastasis
M1 Distant metastasis
M1a Non regional lymph node(s)
M1b Bone(s)
M1c Other site(s)

*Note: When more than one site of metastasis is present, the most advanced category is used.

© 2020 Zumsteg ZS et al. *JAMA Network Open.*

APPENDIX III (con'd)

Histopathologic Grade **(G) (4/5/96)**

**GX**	Grade cannot be assessed
**G1**	Well differentiated, slight anaplasia
**G2**	Moderately differentiated, moderate anaplasia
**G3-4**	Poorly differentiated or undifferentiated, marked anaplasia

STAGE GROUPING

**Stage 0**	T1a	N0	M0	G1
**Stage I**	T1a	N0	M0	G2, G3-4
	T1b, T1c, T1	N0	M0	Any G
**Stage II**	T2	N0	M0	Any G
**Stage III**	T3	N0	M0	Any G
**Stage IV**	T4	N0	M0	Any G
	Any T	N1, N2, N3	M0	Any G
	Any T	Any N	M1	Any G

© 2020 Zumsteg ZS et al. *JAMA Network Open.*

APPENDIX IV

ADVERSE REACTION

REPORTING GUIDELINES A. GENERAL

GUIDELINES

In order to assure prompt and complete reporting of toxicities, the following general guidelines are to be observed. These apply to all RTOG studies and Intergroup Studies in which RTOG participates. **When a protocol toxicity requires special handling, study-specific reporting procedures supercede the General Guidelines.**

1. The Principal Investigator will report the details of any unusual, significant, fatal or life-threatening protocol treatment reaction to the RTOG Group Chairman. In the absence of the Group Chairman, the report should be made to the Headquarters Data Management Staff *(215/574-3214)*. When telephone reporting is required, the Principal Investigator should have all relevant material available. See the protocol-specific criteria to grade the severity of the reaction.

2. The Principal Investigator will also report the details of the significant reaction to the Study Chairman by telephone .

3. A written report containing all relevant clinical information concerning the reported event will be sent to RTOG Headquarters by the Principal Investigator. This must sent within 10 working days of the discovery of the toxicity unless specified sooner by the protocol *(FAX #215/928-0153).*

4. The Group Chairman in consultation with the Study Chairman will take appropriate and prompt action to inform the membership and statistical personnel of any protocol modifications and/or precautionary measures.

5. For those incidents requiring telephone reporting to the National Cancer Institute *(NCI)*, Investigational Drug Branch *(IDB)* or Food and Drug Administration *(FDA)*, the Principal Investigator should first call RTOG *(as outlined above)* unless this will unduly delay the notification process required by the federal agencies.

   A copy of all correspondence submitted to NCI, or to another Cooperative Group *(in the case of RTOG- coordinated intergroup studies)* must also be submitted to RTOG Headquarters when applicable.

6. The Principal Investigator, when participating in RTOG-coordinated Intergroup studies, is obligated to comply with all additional reporting specifications required by an individual study.

7. Institutions must also comply with their individual Institutional Review Board policy with regard to toxicity reporting procedure.

© 2020 Zumsteg ZS et al. *JAMA Network Open.*

8. Failure to comply with reporting requirements in a timely manner may result in suspension of patient registration.

B. RADIATION TOXICITY GUIDELINES

1. All fatal toxicities *(grade 5)* resulting from protocol treatment must be reported by telephone to the Group Chairman, to RTOG Headquarters Data Management and to the primary Study Chairman within 24 hours of discovery.

2. All life-threatening *(grade 4)* toxicities resulting from protocol treatment must be reported by telephone to the Group Chairman, to RTOG Headquarters Data Management and to the primary Study Chairman within 24 hours of discovery.

© 2020 Zumsteg ZS et al. *JAMA Network Open.*

3. Appropriate data forms, and if requested a written report, must be submitted to Headquarters within 10 working days of the telephone report.

C. ADVERSE DRUG REACTIONS- DRUG AND BIOLOGICS

An adverse reaction is a toxicity or an undesirable effect usually of severe nature. Specifically, this may include major organ toxicities of the liver, kidneys, cardiovascular system, central nervous system, skin, bone marrow, or anaphylaxis. These undesirable effects may be further classified as "known" or "unknown" toxicities.

Known toxicities are those which have been previously identified as having resulted from administration of the agent. They may be identified in the literature, the protocol, the consent form or noted in the drug insert.

Unknown toxicities are those thought to have resulted from the agent but have not previously been identified as a known side effect.

Commercial and Non-Investigational Agents

i. Any fatal *(grade 5)* or life threatening *(grade 4)* adverse reaction which is due to or suspected to be the result of a protocol drug must be reported to the Group Chairman or to RTOG Headquarters' Data Management Staff and to the Study Chairman by telephone within 24 hours of discovery. Known grade 4 hematologic toxicities need not be reported by telephone.

ii. Unknown adverse reactions *(≥ grade 2)* resulting from commercial drugs prescribed in an RTOG protocol are to be reported to the Group Chairman or RTOG Headquarters' Data Management, to the Study Chairman and to the IDB within 10 working days of discovery. FDA Form 3500 is to be used in reporting details. All relevant data forms must accompany the RTOG copy of Form 3500.

iii. All neurotoxicities *(≥ grade 3)* from radiosensitizer or protector drugs are to be reported within 24 hours by phone to RTOG Headquarters and to the Study Chairman.

iv. All relevant data forms must be submitted to RTOG Headquarters within 10 working days on all reactions requiring telephone reporting. A special written report may be required.

Reactions definitely thought not to be treatment related should not be reported, however, a report should be made of applicable effects if there is a reasonable suspicion that the effect is due to protocol treatment.

Investigational Agents

Prompt reporting of adverse reactions in patients treated with investigational agents is mandatory. Adverse reactions from NCI sponsored drugs are reported to:

Investigational Drug Branch *(IDB)*
P. O. Box 30012
Bethesda, MD 20824
Telephone number available 24 hours
(301) 230-2330 FAX # 301-230-0159

© 2020 Zumsteg ZS et al. *JAMA Network Open.*

i. *<u>Phase I Studies Utilizing Investigational Agents</u>*

- All deaths during therapy to IDB and with the agent.	Report **by phone** within 24 hours RTOG Headquarters. **A written report to follow within 10 working days.
- All deaths within 30 days above of termination of the agent.	As

29

© 2020 Zumsteg ZS et al. *JAMA Network Open.*

- All life threatening *(grade 4)* As above events which may be due to agent.

- First occurrence of any Report by **phone within 24 hours** to IDB toxicity *(regardless of grade)*. drug monitor and RTOG Headquarters.

  **A written report may be required.

ii. Phase II, III Studies Utilizing Investigational Agents

- All fatal *(grade 5)* and life threatening Report **by phone** to RTOG Headquarters and *(grade 4)* known adverse reactions due to the Study Chairman within 24 hours investigational agent. **A written report must be sent to RTOG

  within working days with a copy to IDB. *(Grade 4 myelosuppression not reported to IDB)*

- All fatal (grade 5) and life threatening Report **by phone** to RTOG Headquarters, the (grade 4) unknown adverse reactions Study Chairman and IDB within **24 hours**. resulting from or suspected to be related **A written report to follow within 10

  to investigational agent. working days.

- All grade 2, 3 unknown adverse reactions **Report **in writing** to RTOG Headquarters and resulting from or suspected to be related IDB within 10 working days.

  to investigational agent.

****** See attached *(if applicable to this study)* NCI Adverse Drug Reaction Reporting Form

© 2020 Zumsteg ZS et al. *JAMA Network Open.*

APPENDIX V

GLEASON CLASSIFICATION1

Histologic patterns of adenocarcinoma of the prostate

Pattern	Margins Tumor Areas	Gland Pattern	Gland Size	Gland Distribution	Stromal Invasion
1	Well defined	Single, separate, round	Medium	Closely packed	Minimal,expansile
2	Less definite	Single,separate rounded but more variable	Medium	Spaced up to one gland diameter, average	Mild, in larger stromal planes
3	Poorly defined	Single,separate more irregular	Small medium, or large	Spaced more than one gland diameter, rarely packed	Moderate, in larger or smaller stromal planes
**or** 3	Poorly defined	Rounded masses of cribriform or papillary epithelium	Medium or large	Rounded masses with smooth sharp edges	Expansile masses
4	Ragged, infiltrating	Fused glandular masses or "hypernephroid"	Small	Fused in ragged masses	Marked,through smaller planes
5	Ragged, infiltrating	Almost absent, few tiny glands or signet ring	Small	Ragged anaplastic masses of epithelium	Severe between stromal fibers or destructive
**or** 5	Poorly defined	Few small lumina in rounded masses of solid epithelium central necrosis	Small	Rounded masses and cords with smooth sharp edges	Expansile masses

The Gleason Classification is a system of histologic grading based on over-all pattern of tumor growth at relatively low-magnification *(40 to 100x)*. Five patterns of growth are recognized and numbered in order of increasing malignancy. Because of

histologic variation in the tumor, two patterns are recorded for each case, a primary or predominal pattern and a secondary or lesser pattern.

The Gleason Score is the sum of the primary and secondary pattern. If only one pattern is present, the primary and secondary pattern receive the same designation.

(Primary = 2, Secondary = 1, Gleason = 3)
(Primary = 2. Secondary = 2, Gleason = 4)

1. Gleason, D.F. et al: Prediction of prognosis for prostatic carcinoma by combined histologic grading and clinical staging. J Urol 111:58, 1974.

© 2020 Zumsteg ZS et al. *JAMA Network Open.*
