# Supplementary material for: Effect of Androgen Deprivation on Long-term Outcomes of Intermediate-Risk Prostate Cancer Stratified as Favorable or Unfavorable: A Secondary Analysis of the RTOG 9408 Randomized Clinical Trial
Source: JAMA Netw Open. 2020 Sep 9;3(9):e2015083. doi: 10.1001/jamanetworkopen.2020.15083 (PMC7489808; doi:10.1001/jamanetworkopen.2020.15083)
Supplement: Supplement 2. — eAppendix. Supplemental Materials and Methods eReferences eFigure. Consolidated Standards of Reporting Trials (CONSORT) of this Secondary Analysis of RTOG 9408 [file jamanetwopen-e2015083-s002.pdf]

Supplementary Online Content

Zumsteg ZS, Spratt DE, Daskivich TJ, et al. Effect of androgen deprivation on long-term outcomes of intermediate-risk prostate cancer stratified as favorable or unfavorable: a secondary analysis of the RTOG 9408 randomized clinical trial. *JAMA Netw Open*. 2020;3(9):e2015083. doi:10.1001/jamanetworkopen.2020.15083

eAppendix. Supplemental Materials and Methods
eReferences
eFigure. Consolidated Standards of Reporting Trials (CONSORT) of this Secondary Analysis of RTOG 9408

This supplementary material has been provided by the authors to give readers additional information about their work.

© 2020 Zumsteg et al. *JAMA Network Open*.

eAppendix: Supplemental Materials and Methods

Patient selection and pretreatment evaluation

Details of patients enrolled in RTOG 9408 (NCT00002597), including inclusion and exclusion criteria, have been published previously.[1] Briefly, patients with clinical stage T1b-T2b prostate adenocarcinoma and a PSA ≤ 20 ng/mL without nodal or distant metastasis were eligible and signed informed consent for the study between October 1994 and April 2001. All patients underwent digital rectal exam, bone scan, and surgical or radiographic evaluation of the pelvic lymph nodes. Those with Karnofsky performance score < 70, alanine aminotransferase more than twice the upper limit of normal, or previous chemotherapy, ADT, radiotherapy, cryotherapy, or definitive prostate cancer surgery were excluded. The primary endpoint was overall survival.

Study design and treatment

Patients were randomized 1:1 to radiotherapy alone or in combination with 4 months of ADT. Patients were stratified according to PSA level (<4 vs. 4-20 ng/mL), tumor grade (well- vs. moderately- vs. poorly-differentiated), and regional lymph node evaluation (pN0: surgical vs. pNx: radiographic). Radiotherapy consisted of 46.8 Gy in 26 fractions to the pelvis, followed by a prostate boost to 19.8 Gy in 11 fractions for a total dose 66.6 Gy in 37 fractions, prescribed to the isocenter. Radiation to the prostate without treatment of the pelvic lymph nodes was allowed for patients with negative lymph node dissections, or PSA < 10 ng/mL and Gleason score ≤5. ADT consisted of combined androgen blockade with flutamide 250 mg three times a day and either goserelin 3.6 mg subcutaneously monthly or leuprolide 7.5 mg intramuscularly monthly, starting 2 months prior to radiation and continuing until radiation completion.

Definition of Favorable vs. Unfavorable Intermediate Risk Prostate Cancer

Unfavorable intermediate risk prostate cancer was defined as a patient with National Comprehensive Cancer Network (NCCN) intermediate risk disease and any of the following: multiple NCCN intermediate risk factors (clinical stage

© 2020 Zumsteg et al. *JAMA Network Open.*

T2b-c, Gleason score =7, PSA = 10-20), Gleason group 3 (4+3=7), or more than 50% of biopsy cores containing cancer. All others were classified as favorable intermediate risk.

Determination of percentage of positive biopsy cores (PPBC)

After obtaining approval from the NRG/RTOG (NRG internal-review board protocol IRB00000781), three physicians (ZSZ, DES, HMS) traveled to NRG Oncology Statistics and Data Management Center in Philadelphia, PA and reviewed digitally archived pathology reports for patients categorized as intermediate-risk in the NCCN classification system, blinded to outcomes. Any ambiguous cases were reviewed by all 3 physicians. Of 1068 intermediate-risk patients, exact positive and total biopsy core numbers were available for 686 (64%). The median number of total biopsy cores was 6 (intraquartile range 6-7) in patients with exact core information. In another 84 patients, although the exact number of cores was not given, it could be clearly determined whether the patient met favorable or unfavorable criteria. These cases usually involved a single positive core among multiple biopsies, or cancer present in all biopsy specimens without the total number given. In another 120 patients, classification as unfavorable intermediate-risk prostate cancer was possible due to the presence of grade group 3 (Gleason 4+3=7) or multiple NCCN intermediate-risk factors, leaving a total of 890 patients (83%) for the analysis of this study.

Endpoints

All endpoints were measured from the date of randomization. Distant metastatic disease was defined as prostate cancer occurring in any anatomic location other than the prostate, seminal vesicles, or pelvic lymph nodes. Prostate cancer-specific mortality (PCSM) was defined per protocol as death directly attributable to prostate cancer, death from treatment complications, or death from unknown causes in patients with active cancers or previously documented clinical or biochemical relapse.

Statistical Methods

© 2020 Zumsteg et al. *JAMA Network Open.*

Baseline clinical characteristics were compared using $\chi 2$ tests for categorical variables and an analysis of variance test for continuous variables. Median follow-up was calculated with the reverse Kaplan-Meier method. The Kaplan-Meier method was used to generate survival curves and to estimate actuarial event-time probabilities for all-cause mortality. The log-rank test was used to compare survival curves. A Cox proportional hazards model was used to generate hazard ratios (HR) and 95% confidence intervals (CI) for OS. The cumulative incidence method was used to estimate PCSM and distant metastasis (DM) at a given time point, with death from causes other than prostate cancer defined as a competing risk. Comparisons of PCSM and DM for different subgroups were performed using a k-sample test for the entire cumulative incidence curves. Multivariate competing risk analysis for PCSM was performed using a Fine and Gray method. All statistical analyses were performed using R version 3.5.1 (R Foundation for Statistical Computing, Vienna, Austria).

eReferences:

1. Jones CU, Hunt D, McGowan DG, et al. Radiotherapy and short-term androgen deprivation for localized prostate cancer. *The New England journal of medicine.* Jul 14 2011;365(2):107-118.

© 2020 Zumsteg et al. *JAMA Network Open.*

eFigure: Consolidated Standards of Reporting Trials (CONSORT) of this Secondary Analysis of RTOG 9408.

© 2020 Zumsteg et al. *JAMA Network Open.*

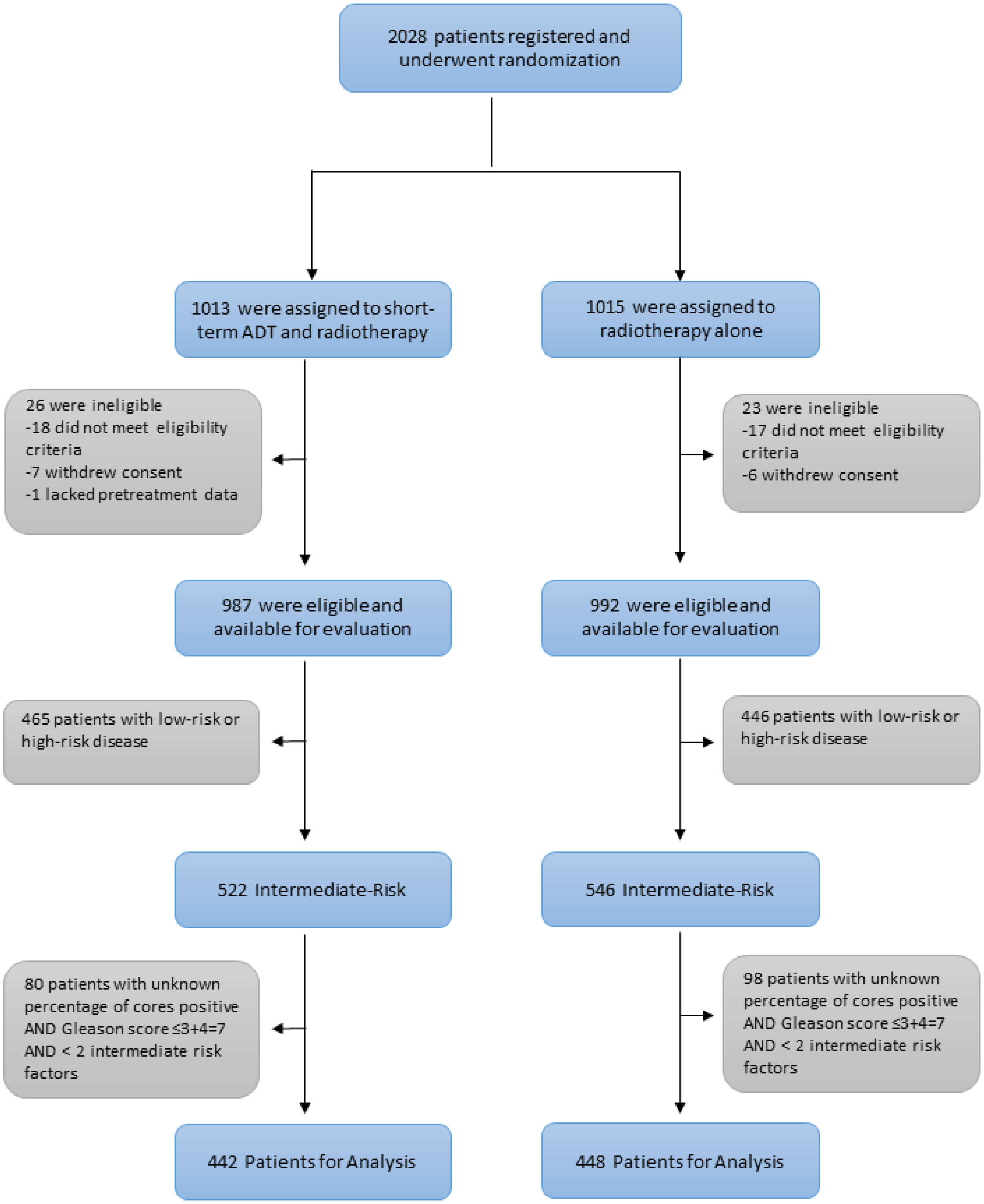

© 2020 Zumsteg et al. *JAMA Network Open.*
