# Supplementary material for: Effect of Androgen Deprivation on Long-term Outcomes of Intermediate-Risk Prostate Cancer Stratified as Favorable or Unfavorable: A Secondary Analysis of the RTOG 9408 Randomized Clinical Trial
Source: JAMA Netw Open. 2020 Sep 9;3(9):e2015083. doi: 10.1001/jamanetworkopen.2020.15083 (PMC7489808; doi:10.1001/jamanetworkopen.2020.15083)
Supplement: Supplement 3. — Data Sharing Protocol [file jamanetwopen-e2015083-s003.pdf]

Data Sharing Statement

Zumsteg. Effect of Androgen Deprivation on Long-term Outcomes of Intermediate-Risk Prostate Cancer Stratified as Favorable or Unfavorable. *JAMA Netw Open*. Published September 09, 2020. 10.1001/jamanetworkopen.2020.15083

Data
Data available: No

Additional Information
Explanation for why data not available: These data can be requested directly from NRG Oncology, but I do not have ownership or permission to release the data outside of the public mechanism for obtaining data from NRG Oncology. Investigators who wish to use data from NRG trials must submit the Ancillary Projects Application Form to NRG (https://www.nrgoncology.org/Resources/Ancillary-Projects-Data-Sharing-Application).
